# The Warburg effect is necessary to promote glycosylation in the blastema during zebrafish tail regeneration

**DOI:** 10.1038/s41536-021-00163-x

**Published:** 2021-09-13

**Authors:** Jason W. Sinclair, David R. Hoying, Erica Bresciani, Damian Dalle Nogare, Carli D. Needle, Alexandra Berger, Weiwei Wu, Kevin Bishop, Abdel G. Elkahloun, Ajay Chitnis, Paul Liu, Shawn M. Burgess

**Affiliations:** 1 Translational and Functional Genomics Branch, National Human Genome Research Institute, Bethesda, MD USA; 2 Aquatic Models of Human Development Affinity Group, National Institute of Child Health and Human Development, Bethesda, MD USA; 3 Cancer Genetics and Comparative Genomics Branch, National Human Genome Research Institute, Bethesda, MD USA

**Keywords:** Regeneration, Gene regulation

## Abstract

Throughout their lifetime, fish maintain a high capacity for regenerating complex tissues after injury. We utilized a larval tail regeneration assay in the zebrafish *Danio rerio*, which serves as an ideal model of appendage regeneration due to its easy manipulation, relatively simple mixture of cell types, and superior imaging properties. Regeneration of the embryonic zebrafish tail requires development of a blastema, a mass of dedifferentiated cells capable of replacing lost tissue, a crucial step in all known examples of appendage regeneration. Using this model, we show that tail amputation triggers an obligate metabolic shift to promote glucose metabolism during early regeneration similar to the Warburg effect observed in tumor forming cells. Inhibition of glucose metabolism did not affect the overall health of the embryo but completely blocked the tail from regenerating after amputation due to the failure to form a functional blastema. We performed a time series of single-cell RNA sequencing on regenerating tails with and without inhibition of glucose metabolism. We demonstrated that metabolic reprogramming is required for sustained TGF-β signaling and blocking glucose metabolism largely mimicked inhibition of TGF-β receptors, both resulting in an aberrant blastema. Finally, we showed using genetic ablation of three possible metabolic pathways for glucose, that metabolic reprogramming is required to provide glucose specifically to the hexosamine biosynthetic pathway while neither glycolysis nor the pentose phosphate pathway were necessary for regeneration.

## Introduction

Many nonmammalian vertebrates have the ability to regenerate complex appendages following loss or injury. Appendage regeneration is a process involving the coordinated interaction of multiple cell types following injury: there is an immune response and the establishment of a wound epithelium or a specialized epithelial structure known as the apical epidermal cap (AEC), and then signaling that emanates from the newly formed wound epithelium/AEC through growth factors such as TGF-β and FGF. These early responses promote cell migration and proliferation, and are required for blastema formation, the key step leading to complete tissue restoration of an appendage. The blastema, which has been described as mesenchymal in nature, is a mass of multipotent cells capable of proliferating and differentiating to replace lost tissue. Although mammals typically have a very limited capacity to form blastemas after injury and thus cannot regenerate appendages, there are a few examples where blastema formation can occur in mammals. Two species of African spiny mouse have been reported to form blastema-like structures during skin shedding^1^ and digit tip regeneration in mice also involves the formation of a blastema from fate-restricted progenitors^2^. Human children are also capable of digit tip regeneration, suggesting limited blastema formation is at least possible in humans^3^. Interestingly, in contrast to most commonly studied mouse strains, the MRL mouse does form a blastema around ear punctures, suggesting that tissue regeneration in mammals could be enhanced or even initiated with a better understanding of the pathways underlying blastema formation^4^. Although progress has been made in determining cellular signals required for blastema formation and maintenance, our understanding of the mechanisms by which apparently differentiated cells dedifferentiate to become the blastemal mesenchyme remains incomplete.

A frequently observed phenomenon during regeneration is metabolic reprogramming in some cells, or the uncoupling of glucose metabolism from oxidative phosphorylation. In addition to increased glycolysis, uncoupling can also result in glucose being shunted into two metabolic pathways that branch off from glycolysis, the pentose phosphate pathway (PPP) and the hexosamine biosynthetic pathway (HBP). Metabolic shifting in glucose metabolism has been proposed as a mediator of early tail regeneration in both *Xenopus* and lizards^5,6^ and has been shown to be essential in zebrafish heart regeneration^7^. Beyond regeneration, there are many other examples of metabolic reprogramming regulating cellular function. For example, human embryonic stem cells rely on glycolysis in a mildly hypoxic environment for self-renewal. Upon exposure to oxygen, these cells increase oxidative phosphorylation and quickly lose pluripotency^8^. Inflammatory macrophages rely primarily on glycolysis and the PPP while repair macrophages utilize oxidative phosphorylation and switching in these cells activates distinct transcriptional profiles^9,10^. Glucose shunting to the HBP and PPP determines cell fate between trophectoderm precursors and the inner cell mass during embryogenesis^11^. Perhaps the most well known and studied example of metabolic reprogramming is the Warburg effect in cancer cells which is defined by increased glucose uptake and lactate production. Although the purpose of the Warburg effect is highly debated, proposed reasons include faster, albeit less efficient ATP production, adaption to hypoxic conditions, shunting of glucose metabolites to branched pathways, manipulation of the microenvironment, and metabolites used for cell signaling^12^. With respect to cell signaling, it is becoming increasingly clear that metabolic reprogramming plays a role in promoting an epithelial to mesenchyme transition (EMT) in tumor cells^13^, a process that has been associated with the development of cancer stem cells^14^. A metabolic switch to glycolysis has also been demonstrated in dedifferentiated human cells^15^. Taken together with what has been observed during regeneration, metabolic reprogramming may be a general feature of dedifferentiating cells.

It has been shown that the zebrafish *Dario rerio* can regenerate many tissues after injury or amputation including the fins^16^, the heart^17^, and the spinal cord^18^. In zebrafish embryos, tail amputation through the posterior trunk of the animal results in repositioning of notochord cells to the amputation site, followed by blastema formation and regeneration of several different tissues including the notochord, the neural tube, blood vessels, and the caudal fin-fold^19^. An advantage to using the embryonic tail over the adult fin is that many genes required for regeneration are essential for long term viability of the animal^20^. Studying early embryos circumvents the need for conditional mutations. Using this tail excision model, previous reports have shown that sonic hedgehog and EGFR signaling are required for regeneration^19,21^. However, the full genetic pathway for blastema formation following repositioning of notochord cells has not been determined. Here, we show that metabolic reprogramming to promote glucose flux through the HBP and N-linked glycosylation is obligate for tail regeneration, but glycolysis and the PPP are not. Blocking glucose metabolism with the glucose analog 2-deoxyglucose (2-DG) resulted in a complete failure of the tail to regenerate following amputation but had no gross effects on early larval development under normal conditions. During early regeneration, we observed increased glucose uptake and an altered mitochondrial morphology and oxidation state within the notochord bead of the amputated tail, indicative of metabolic reprogramming. Single-cell RNA-seq (scRNA-seq) analyses revealed that 2-DG treatment resulted in the failed induction of TGF-β ligands essential to triggering blastema formation while simultaneously inducing ER stress, indicating disruption of the secretory pathway. Accordingly, genetic evidence indicated that while glycolysis and the PPP are dispensable for tail regeneration, the HBP is absolutely required. Moreover, mutations in *gfpt1*, which encodes the rate-limiting enzyme of the HBP, resulted in hypersensitivity to 2-DG during regeneration, further demonstrating that the specific purpose of shifting to aerobic glycolysis was to promote glucose flux through the HBP and not for ATP generation by glycolysis. Finally, direct inhibition of N-linked glycosylation with tunicamycin also resulted in impaired regeneration. Together our data indicate that a metabolic shift to promote glycosylation is a key event regulating normal blastema formation through triggering signaling pathways such as TGF-β, resulting in expression of mesenchymal genes such as *snai1a* and *msx3*. The metabolic shift to promote glycosylation that takes place during blastema formation suggests potential parallels between tissue regeneration and tumor biology, including ways to regulate or inhibit them to our advantage.

## Results

### Tail regeneration following amputation

**Fig. 1:**
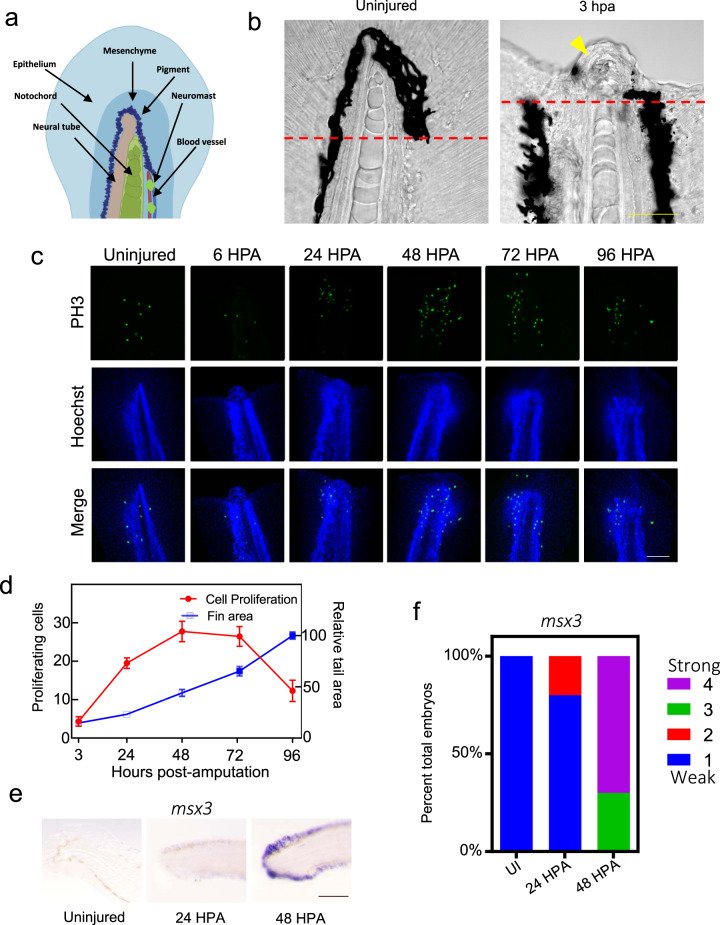
Tail regeneration following amputation in zebrafish embryos. **a** An illustration showing tissues that are regenerated following tail amputation. **b** Left, a normal tail fin with the amputation plane indicated with a dashed red line. Right, an image depicting the notochord bead formation (yellow arrowhead) at 3 HPA. Embryos are 3 DPF. Scale bar = 50 µm. **c** Tail images of PH3^+^ following tail amputation at indicated timepoints in hours post-amputation. Uninjured embryos are 4 DPF. Amputations were performed at 3 DPF. Images are sum z-stack projections of 1 µM slices through the entire embryo. Scale bar = 100 µm. **d** Quantification of cell proliferation as determined by PH3^+^ cells and tail area during regeneration. For tail area, *N* = 10 at all timepoints. For proliferation, *N* = 10, 10, 8, 9, and 7 for 3, 24, 48, 72, and 96 HPA, respectively. Mean and SEM are shown. **e** In situ hybridization of the blastema marker *msx3* in uninjured animals and at 24 or 48 HPA. Scale bar = 100 µm. **f** Stacked percentage graph of *msx3* expression as determined by in situ hybridization. A score of 1 represents little to no staining, while a score of 4 represents strong staining. *N* = 10 embryos for all samples.

Deep amputation through the posterior trunk, or the tail, of the larval zebrafish results in regeneration of several types of tissue including the notochord, neural tube, blood vessels, and the caudal fin-fold (Fig. 1a). Following amputation, the epithelium closes within one hour (Supplementary Fig. 1a, b). Concurrently, notochord cells migrate to the injury plane and form a small “bead” (Fig. 1b), a step necessary to initiate cell proliferation^19^. Utilizing a double transgenic zebrafish line, Tg(*col8a1a*:GFP; *col9a2*:mCherry), which expresses GFP in the vacuolated notochord cells and mCherry in the surrounding sheath cells^22^, we could show both cell types repositioned into the notochord bead post-amputation, generally maintaining the notochord architecture with vacuolated cells located on the interior of the bud surrounded by sheath cells (Supplementary Fig. 1c, Supplementary Movie 1). Therefore, *col9a2*:mCherry can be used as a marker for the notochord bead. To gain a better understanding of cell proliferation both spatially and temporally during tail regeneration, we utilized an antibody against the proliferation marker phospho-histone H3 (PH3). We observed an increase in cell proliferation by 24 HPA compared to uninjured tails, with proliferation peaking between 48 and 72 HPA before decreasing by 96 HPA (Fig. 1c, d). A second marker of proliferation, the dual Fucci transgenic zebrafish that expresses cerulean in cells within the S, M, or G2 phases and mCherry in cells in G0/1, also showed an increase in proliferating cells post-amputation, although in this line proliferation peaked between 24 and 48 HPA (Supplementary Fig. 1d, e)^23^. In agreement with previous reports, the blastema is evident by 48 HPA, as shown by strong induction of the blastema/mesenchyme marker *msx3* (Fig. 1e, f, Supplementary Fig. 1f). Fin area steadily increased throughout the time-course, irrespective of the number of proliferating cells present, indicating that cell migration, proliferation, differentiation, and hypertrophic cell growth all likely contribute to fin regrowth between 3 and 96 HPA (Fig. 1d).

### A metabolic shift to glucose metabolism is essential for regeneration

**Fig. 2:**
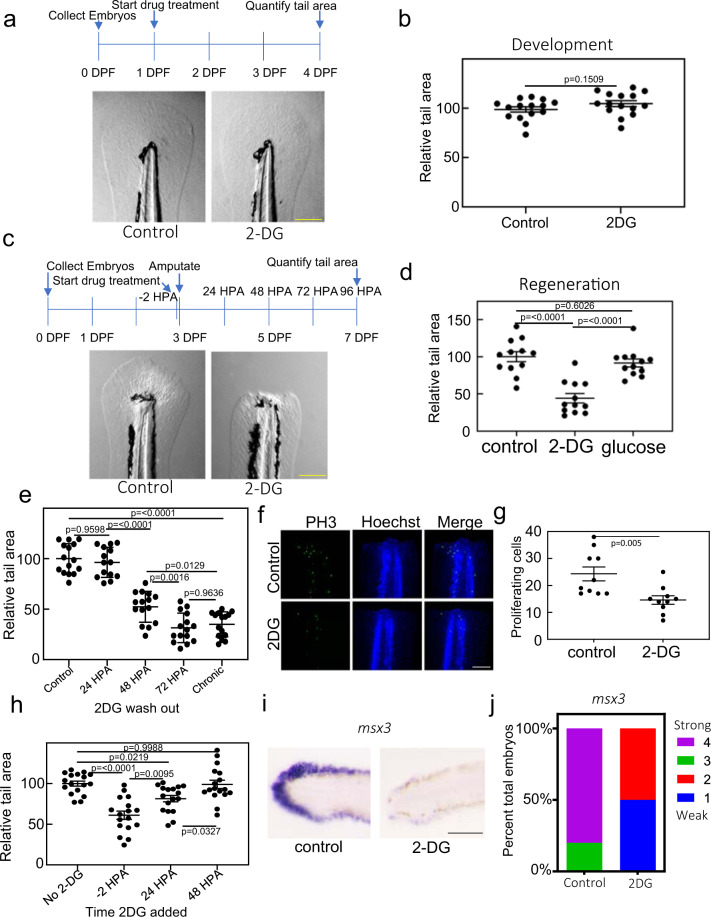
Blocking glucose metabolism inhibits tail regeneration. **a** Images of 4 DPF embryo tails. Embryos were untreated (control) or treated with 2-DG from 1 DPF throughout the duration of the experiment. Scale bar = 200 µm. **b** Quantification of tail surface area of untreated or 2-DG treated embryos. Mean and SEM are shown. *N* = 15 embryos per samples. Statistics were determined using an unpaired *t*-test. **c** Images of 7 DPF embryo tails 4 days post amputation. Embryos were untreated (control) or treated with 2-DG from 2 h before the amputation and throughout the duration of the experiment. Scale bar = 200 µm. **d** Quantification of tail surface area of untreated, 100 mM 2-DG, or 100 mM glucose treated embryos 96 HPA. *N* = 12 embryos for all samples. Statistics were determined by one-way ANOVA. Mean and SEM are shown. **e** Quantification of tail surface area of control or 2-DG treated embryos 96 HPA. *X*-axis indicates timepoint that 2-DG was washed out of media. Mean and SEM are shown. *N* = 14–15 embryos for all samples. Statistics were determined by one-way ANOVA. **f** Confocal images of PH3 staining in untreated and 2-DG treated embryo tails at 24 HPA. Images are sum z-stack projections of 1 µM slices through the entire embryo. Scale bar = 100 µm. **g** Quantification of proliferating cell as determined by PH3 staining. Mean and SEM are shown. *N* = 10 embryos for all samples. Statistics were determined by an unpaired two-tailed *t*-test. **h** Quantification of the tail surface area for untreated or 2-DG treated embryos at 96 HPA. *X*-axis indicates the timepoint that 2-DG was added to the media. Mean and SEM are shown. *N* = 20 embryos per sample. Statistics were determined by one-way ANOVA. **i** In situ hybridization of *msx3* in untreated and 2-DG treated embryos at 48 HPA. Scale bar = 100 µm. **j** Stacked percentage graph of *msx3* expression as determined by in situ hybridization. A score of 1 represents little to no staining, while a score of 4 represents strong staining. *N* = 10 embryos for all samples.

Metabolic switching has been shown to play a key role in immune cell responses^24^, stem cell maintenance^8,25^, and cancer progression^26^. To understand if it also has a role in regeneration, we impaired either mitochondrial function or glucose metabolism during development and tail regeneration. We treated 1-day post-fertilization (DPF) embryos with the mitochondrial import inhibitor mitoblock-6 (MB-6), which has been shown to decrease respiration^27^. Treatment resulted in severe developmental abnormalities as previously reported (Supplementary Fig. 2a)^28^. We also observed a reduction in tail size, consistent with an essential role for respiration during early development (Supplementary Fig. 2b, c). In contrast, 2-DG, a nonmetabolizable glucose analog and well-described glycolysis inhibitor, had no obvious gross effect on embryo development, and we observed no significant differences in tail size between control and 2-DG treated animals (Fig. 2a, b and Supplementary Fig. 2d). This in agreement with a previous report, in which it was found that 2-DG treatment or mutation of the glycolysis enzyme *pgk1* resulted in impaired hair cell and neuron development but no major gross early developmental abnormalities^29^. It is unlikely the lack of gross effect on development was due to 2-DG not getting into the embryo. Glucose transporters have been shown to be expressed in the zebrafish larval skin^30^. Additionally, embryos pulsed with the fluorescent glucose analog 2-(N-(7-Nitrobenz-2-oxa-1,3-diazol-4-yl)Amino)-2-Deoxyglucose (2-NBDG), which like 2-DG is imported via glucose transporters and has been shown to be a reliable analog of glucose uptake in zebrafish embryos^31–33^, showed fluorescence throughout the body, indicating that exogenously supplied glucose analogs are efficiently imported into tissues throughout the developing embryo (Supplementary Fig. 2e). Moreover, 2-DG has been shown to suppress lactate production in uninjured zebrafish embryos, and in support of our results no gross morphological defects were observed^34^. Next, we amputated tails of embryos treated with MB-6. MB-6 treated embryos that survived until 7 DPF had a significant but modest reduction in fin size following amputation, proportional to the decreased fin size that blocking mitochondrial function had during development (Supplementary Fig. 2f, g). Despite the slightly reduced size, MB-6 treated fins appeared to regenerate essentially normally as evidenced by a similar morphology to control fins and formation of a relatively normal blastema (Supplementary Fig. 2h, i). However, in larvae treated with 2-DG, fins completely failed to regenerate compared to control animals (Fig. 2c, d). Treatment with comparable concentrations of glucose had no effect on regeneration, indicating that this block was specific to 2-DG (Fig. 2d) and not a result of changes in the osmolarity of the surrounding media. Washing out 2-DG prior to 72 HPA resulted in a partial to complete reinitiation of fin regeneration depending on prior duration of treatment, indicating that the effect of 2-DG treatment was reversible and that 2-DG continued to be taken up by the animal after closure of the wound epithelium (Fig. 2e). Additionally, 2-DG treatment resulted in a decrease of proliferating cells in the regenerating tail (Fig. 2f, g, Supplementary Fig. 2j, k). Addition of 2-DG to embryo media at 24 HPA, well after closer of the wound epithelium, still results in a significant impairment of regeneration, indicating that the effect of 2-DG on regeneration is not simply due to excess 2-DG getting into wound prior to closure of the epithelium (Fig. 2h). However, addition of 2-DG after 48 HPA resulted in no phenotype, indicating that there is an obligate metabolic shift early in the regenerative process (Fig. 2h). This shift is necessary prior to blastema formation (Fig. 1e, f) but it is not necessary in later stages of regeneration even though there is still significant cell division taking place (Fig. 1d), calling into question the argument that a shift to aerobic glycolysis is to facilitate rapid cell division. In situ hybridization of the blastema marker *msx3* showed that unlike inhibition of mitochondrial function, inhibition of glucose metabolism resulted in the loss of normal blastema formation, indicating that metabolic reprogramming is absolutely required for normal blastema development (Fig. 2i, j). The lack of blastema development was not due to increased apoptosis, suggesting that 2-DG inhibited blastema formation through another mechanism (Supplementary Fig. 2l). Together, these results indicate that while oxidative phosphorylation is essential for normal development and glucose metabolism is not for most tissues, there is an obligate shift to promote glucose metabolism during the early stages of blastema formation and tail regeneration.

### Amputated tails display increased glucose uptake and hyperoxidized, fragmented mitochondria

**Fig. 3:**
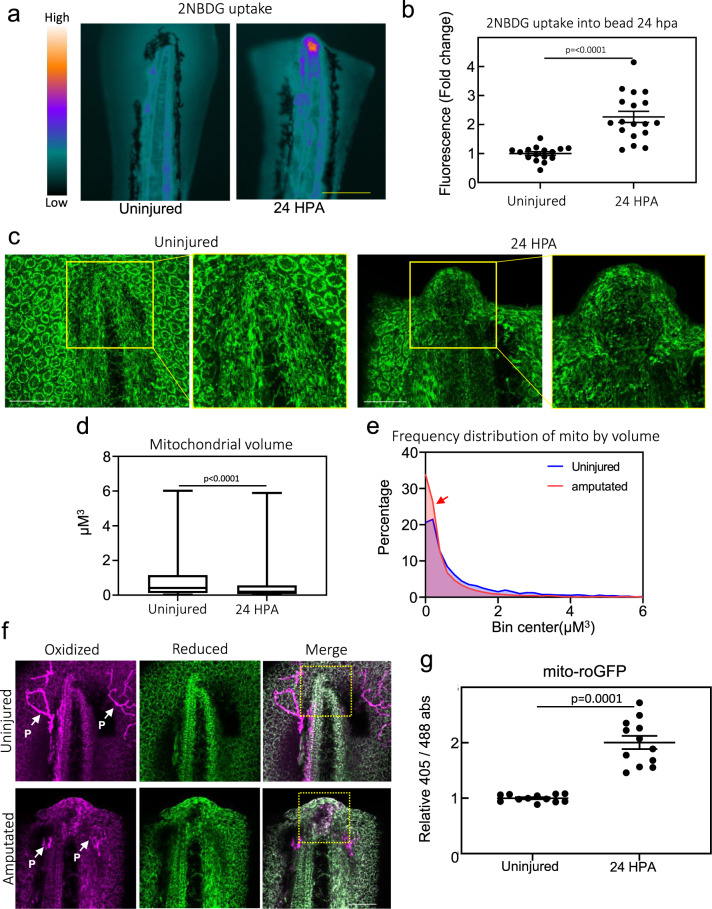
Metabolic reprogramming during early regeneration. **a** Heatmap of fluorescent intensity of uninjured and 24 HPA embryos pulsed with 500 µM 2-NBDG for 2 h. Scale bar = 200 µm. **b** Quantification of 2-NBDG fluorescence in tail of uninjured embryos and notochord bead of 24 HPA embryos. Mean and SEM are shown. *N* = 18–20 embryos per condition. Statistics were determined with an unpaired two-tailed *t*-test. **c** Confocal images of uninjured or 24 HPA Tg(*actb2*:mito-GFP) embryos. Right panels are zoomed-in images of boxed region in left panels. Scale bar = 50 µm. Images are sum z-stacks of 13 1 µM slices excluding epithelium above and below the notochord and notochord bead on the *Z*-axis. **d** Box and whisker plot of mitochondrial volume quantified from the notochord and notochord bead of uninjured and 24 HPA embryos, respectively. Boxes show the 25–75th percentiles, whiskers show the min and max. Lines in the middle of the boxes are the median. N for uninjured sample is 2914 mitochondria from 5 embryos. N for 24 HPA sample is 6791 mitochondria from 5 embryos. Statistics were determined with an unpaired two-tailed *t*-test. **e** Histogram of mitochondrial volume quantified from the notochord and notochord bead of uninjured and 24 HPA embryos, respectively. Red arrow indicates increased numbers of small mitochondria after amputation. N for uninjured sample is 2914 mitochondria from 5 embryos. N for 24 HPA sample is 6791 mitochondria from 5 embryos. **f** Confocal images of uninjured or 24 HPA Tg(*actb2*:mito-roGFP) embryos. Images are sum z-stacks of 17 1 µM slices excluding epithelium above and below the notochord and notochord bead on the *Z*-axis. P indicates auto-fluorescent pigment cells which are not depicting roGFP2. A region of oxidized mitochondria can be seen in amputated tails, depicted by the boxed region, which is not seen in a corresponding region of unamputated tails. Scale bar = 50 µm. **g** Quantification of relative absorbance of roGFP2 at 488 or 405 nm in the notochord bead of 24 HPA embryos or tail of uninjured embryos. *N* = 12 embryos per condition. Mean and SEM are shown. Statistics were determined with an unpaired two-tailed *t*-test.

In order to understand if glucose uptake was increased during regeneration, we pulsed uninjured and 24 HPA embryos with 2-NBDG. Increased uptake was primarily observed in the notochord bead on the stump of the regenerating tail of 24 HPA embryos (Fig. 3a, b, Supplementary Fig. 3a). This increase in 2-NBDG uptake was not simply due to a disruption in the epithelium, as previously mentioned the epithelium closes within 1 h after amputation (Supplementary Fig. 1a, b).

Cancer cells exhibiting a Warburg-like physiology have increased mitochondrial fragmentation compared to cells utilizing oxidative phosphorylation^35^, therefore we analyzed mitochondrial morphology by generating a transgenic zebrafish line, Tg(*actb2*:MLS-EGFP), which expressed GFP with the Cox VIII mitochondrial localization sequence (MLS) fused to the N-terminus thereby making the mitochondria fluorescent^36^. Consistent with previous reports, the Cox VIII specifically targeted GFP to the mitochondria (Supplementary Fig. 3b). Interestingly, we observed fragmented mitochondria within the notochord bead, further indicating that metabolic reprogramming was taking place during early tail regeneration (Fig. 3c, d, e, Supplementary Fig. 3c). In addition to fragmented mitochondria, cancer cells can also show an increase in mitochondrial ROS^37^. To probe mitochondrial ROS production, we generated a second mitochondrial reporter line, Tg(*actb2*:MLS-roGFP2), expressing a redox sensitive GFP (Supplementary Fig. 3b). We observed an increase in the oxidization state of mitochondria, possibly due to increased ROS production, specifically within and directly anterior to the notochord bead, similar to the regions of increased glucose uptake and mitochondrial fragmentation (Fig. 3f, g, Supplementary Fig. 3d)^38^. This shift in absorbance was not observed in Tg(*actb2*:MLS-GFP) embryos, indicating the effect was specific to redox sensitive roGFP2 (Supplementary Fig. 3e). These results are consistent with the observation that there is increased lactate production in the notochord bead during regeneration, indicative of the Warburg effect^34^. Taken together, these data demonstrate that a metabolic shift to aerobic glycolysis takes place in the regenerating tail prior to blastema development.

### 2-DG suppresses normal blastema development

**Fig. 4:**
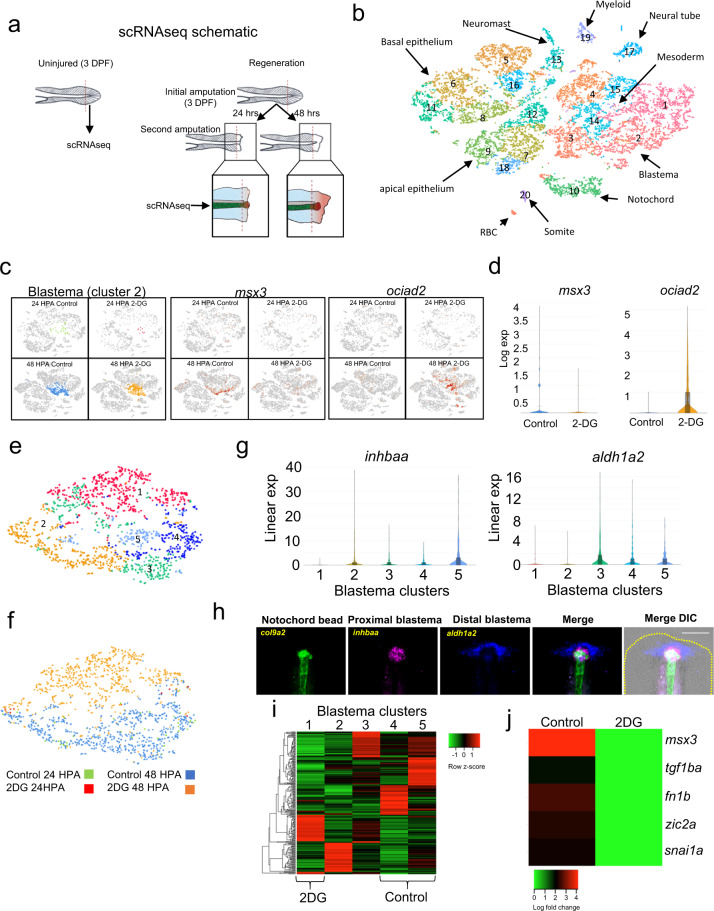
2-DG prevents formation of normal blastema. **a** Illustration depicting the region of uninjured and regenerating tail taken for scRNA-seq. **b** t-SNE plot generated from Louvain clustering of cells from the scRNA-seq data. Number indicates cluster number. **c** Expression of *msx3* and *ociad2* transposed onto the t-SNE plot of cells from the scRNA-seq, shown by library. **d** Violin plots of *msx3* and *ociad2* expression in control and 2-DG treated blastemas (cluster 2). **e** t-SNE plot of reclustered blastema. Numbers indicate cluster number. **f** t-SNE plot of blastema clusters depicted by library. **g** Violin plots of *inhbaa* and *aldh1a2* expression in the reclustered blastema clusters. **h** RNA-FISH images of *col9a2*, *inhbaa*, and *aldh1a2*, which depict the notochord / notochord bud, proximal blastema and distal blastema, respectively, in a 48 HPA embryo. Yellow dashed line indicates outline of tail. Scale bar = 100 µm. **i** Heatmap of marker genes for each reclustered blastema cluster. Cluster 4 and 5 are almost exclusively comprised of untreated control cells while cluster 1 is almost exclusively comprised of 2-DG treated cells. **j** Heatmap of expression of mesenchymal and blastema genes suppressed by 2-DG treatment.

Our data indicated that cells in the regenerating tail undergo metabolic modifications consistent with increased glucose flux through glycolytic or related pathways early in the regenerative process, and this shift is essential for tail regeneration, at least in part, through blastema formation. In order to understand how metabolic reprogramming is involved in triggering regeneration, we performed scRNA-seq on uninjured, 24 HPA and 48 HPA tails with and without 2-DG treatment (Fig. 4a, Supplementary Fig. 4a). Based on gene expression in the untreated samples, we were able to identify specific populations of cells consistent with cell types expected to be present in the regenerating tail (Fig. 4b, Supplementary Fig. 4b, Supplementary Data 1). One cluster that was almost exclusively unique to the 48 HPA library, corresponding to blastema formation, was cluster 2 (Supplementary Fig. 4c). The most significant gene distinguishing this cluster was *aldh1a2*, a well-described blastema marker in both this model and others (Supplementary Data 1)^19,39,40^. This cluster was also enriched for other blastema expressed genes such *msx3*, *loxa*, *zic2*, and *tnc* (Supplementary Data 1)^41^. Therefore, we concluded that cluster 2 was the blastema. Visualizing our scRNA-seq of regenerating tails as a t-SNE plot by library showed that control and 2-DG treated cells formed distinguishable groups within the blastema cluster, indicating that 2-DG strongly altered gene expression within the blastema (Supplementary Fig. 4d). Among the genes significantly suppressed by 2-DG was *msx3*, confirming our previous data and analyses suggesting that the blastema does not form properly when glycolysis is inhibited (Fig. 4c, d). However, several genes were upregulated by 2-DG in the blastema, including the tumor suppressor *ociad2* (Fig. 4c, d, and Supplementary Data 2). To further analyze the effect of 2-DG on blastema formation, we specifically subclustered the blastema cells. This resulted in splitting the blastema into 5 clusters (Fig. 4e, Supplementary Data 3), with cells almost entirely grouped by treatment (Fig. 4f). Cluster 2 and 5 were enriched for *inhbaa*, while cluster 3 and 4 were enriched for *aldh1a2* (Fig. 4g). Visualization of expression of these genes in the tail by fluorescent in situ hybridization suggested that *inhbaa* and *aldh1a2* marked the proximal and distal regions of the blastema with respect to the notochord bead (*col9a2*), respectively (Fig. 4h). When treated with 2-DG, an aberrant *ociad*^+^ blastema cluster (cluster 1) is formed which shows markedly altered gene expression when compared to normal blastema (Fig. 4e, i). Genes suppressed in the *ociad2*^+^ cluster 1 compared to the mainly control clusters 4 and 5 include genes involved in mesenchymal formation (*tgfb1a*, *fn1b*, *snai1a*) and the blastema genes *msx3* and *zic2a* (Fig. 4j). Therefore, 2-DG treatment prevents the formation of normal mesenchymal blastema. While 2-DG did result in changes to gene expression in uninjured animals, further evidence that it can indeed be taken up by uninjured animals prior to the feeding stage, subclustering of fin-fold mesenchymal cells from these animals did not result in separate clusters of control and 2-DG treated cells, indicating 2-DG had a stronger effect in the blastema mesenchyme than the fin-fold mesenchyme (Supplementary Fig. 4e, Supplementary Data 4). Together, our data show that blocking glucose metabolism prevents formation of the blastemal mesenchyme.

### TGF-β signaling is required for blastema formation

**Fig. 5:**
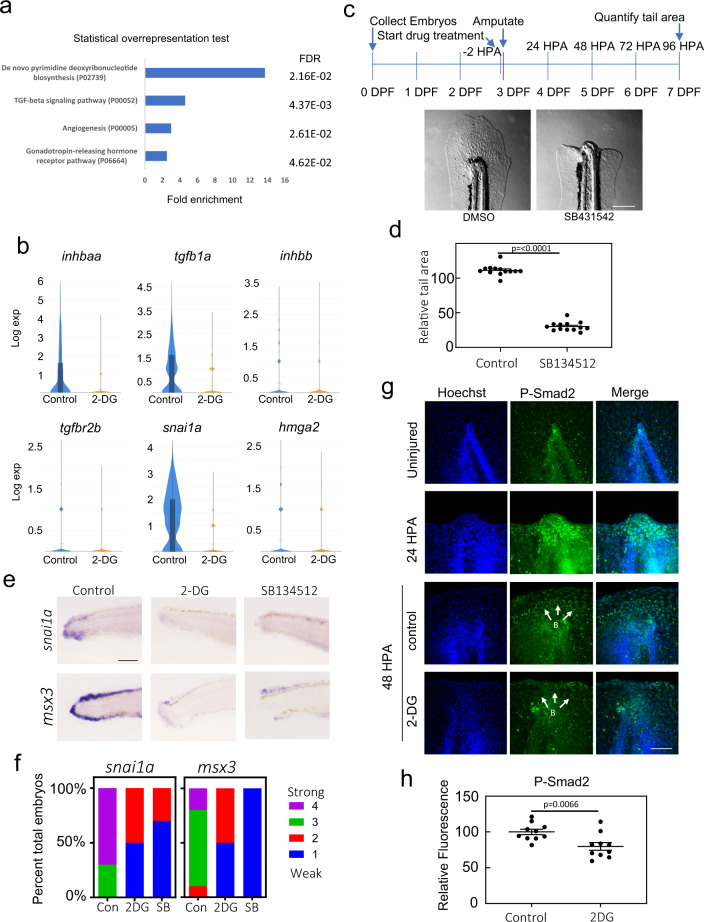
TGF-β signaling is essential for development of blastema. **a** Panther Pathways significantly overrepresented by genes repressed by 2-DG in the blastema (Cluster 2). **b** Violin plots of *tgfb1, inhbaa, inhbb, tgfbr2b, snai1a*, and *hmga2* expression in control and 2-DG treated blastemas (cluster 2). **c** Image of the tail of a 96 HPA embryo treated with DMSO or the Alk4, 5, and 7 inhibitor SB431542. Drug was added to embryo media 2 h prior to amputations and treatment persisted through the duration of experiment. Scale bar = 200 µm. **d** Quantification of tail area of 96 HPA embryos treated with DMSO or SB431542. Drug was added to embryo media 2 h prior to amputations and treatment persisted throughout the duration of experiment. Mean and SEM are shown. *N* = 13 for each condition. Statistics were determined with an unpaired two-tailed *t*-test. **e** In situ hybridization of *snai1a* and *msx3* in DMSO, 2-DG, or SB431542 treated 48 HPA embryos. Scale bar = 100 µm. **f** Stacked percentage graph of *snai1a* and *msx3* expression as determined by in situ hybridization. A score of 1 represents little to no staining, while a score of 4 represents strong staining. *N* = 10 embryos for all conditions. **g** Immunofluorescent staining of P-SMAD2 in uninjured, 24 HPA, and 48 HPA control or 2-D treated embryos. Images are sum z-stack projections of 1 µM slices through the entire embryo. B indicates predicted blastema. Scale bar = 50 µm. **h** Quantification of P-Smad2 intensity in the blastema. Mean and SEM are shown. *N* = 10 embryos for all conditions. Statistics were determined with an unpaired two-tailed *t*-test.

In order to understand how inhibition of glucose metabolism results in aberrant blastema formation, we examined pathways that were significantly overrepresented among genes suppressed by 2-DG in the blastema. As expected because of the reduced cell division in 2-DG treated cells (Fig. 2f, g), genes in the de novo pyrimidine deoxyribonucleotide biosynthesis pathway, which is important for DNA replication and therefore cell proliferation, are overrepresented (Fig. 5a). However, genes involved in TGF − β signaling, a well-characterized pathway in regeneration, were also significantly overrepresented. TGF − β pathway genes significantly suppressed by 2-DG included ligands *tgfb1a*, *inhbaa*, *inhbb*, and the receptor *tgfb2a* (Fig. 5b, Supplementary Data 2). Additionally, known TGF − β target genes *snai1a* and *hmga2* were suppressed by 2-DG. Accordingly, inhibition of TGF − β signaling with SB134512 blocked regeneration (Fig. 5c, d) consistent with observations seen in adult tail fin regeneration^42^, and similar to 2-DG treatment, resulted in repression of *snai1a* and *msx3* (Fig. 5e, f). Also similar to 2-DG treatment, immunofluorescence analyses of PH3 suggested that cell proliferation was reduced in the presence of the TGF − β inhibitor, although this effect was not observed in Fucci fish (Supplementary Fig. 5a, b, c, d). Together, our data indicate that TGF − β signaling is necessary for formation of blastemal mesenchyme.

To examine whether the transcriptional suppression of TGF − β genes by 2-DG reflected an actual reduction in TGF − β signaling, we performed immunofluorescence using antibodies to phospho-SMAD2 (P-SMAD2). Immunofluorescence revealed strong activation of TGF − β signaling in the notochord bead by 24 HPA, followed by signaling in the blastema at 48 HPA (Fig. 5g, Supplementary Fig. 5e). As expected, activation was reduced with the TGF − β inhibitor SB431542, indicating the P-SMAD2 antibody staining was specific for TGF − β signaling (Supplementary Fig. 5f, g). At 48 HPA, we observed a decrease in nuclear P-SMAD2 staining in the blastema upon 2-DG treatment, consistent with repression of TGF − β ligand transcription in the blastema (*inhbaa*, *inhbb*, *tgfb1*) (Fig. 5g, h). Together, our data indicate that metabolic reprogramming was necessary to sustain TGF − β signaling, which promotes blastema formation.

### Genetic perturbation of the hexosamine biosynthetic pathway phenocopies 2-DG treatment

**Fig. 6:**
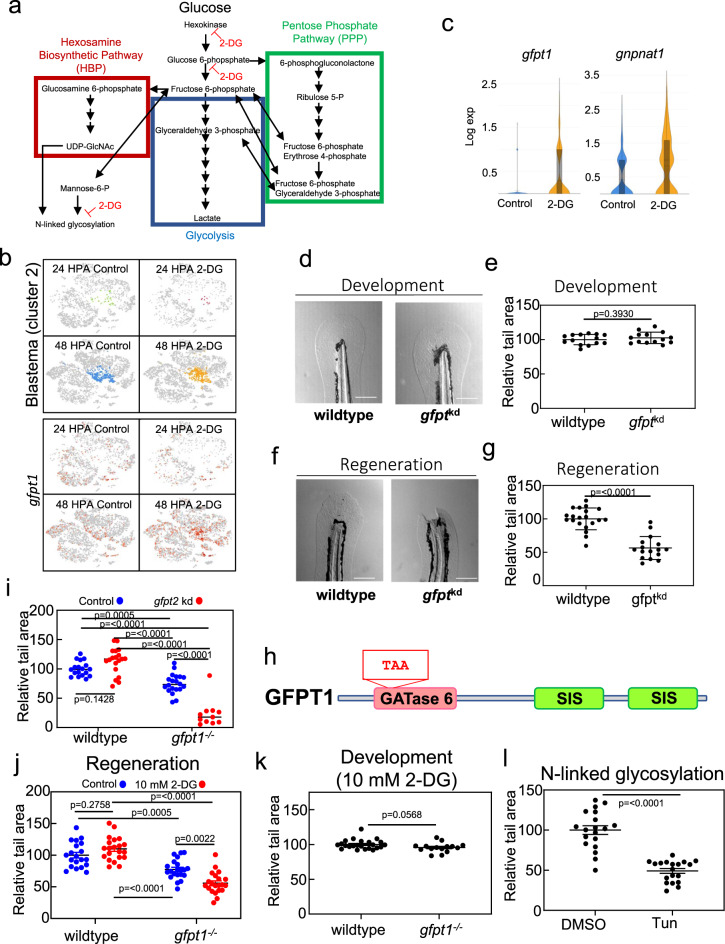
The HBP and N-linked glycosylation trigger tail regeneration. **a** Diagram depicting the HBP (red), glycolysis (blue), and the PPP (green). Single-head Arrows indicate pathway intermediates/products. Double-headed arrows indicate shared pathway substrates. Points where 2-DG acts as an inhibitor are indicated. **b** Expression of *gfpt1* transposed onto the t-SNE plot of cells from the scRNA-seq, shown by library. **c** Violin plots of *gfpt1* and *gnpnat1* expression in control and 2-DG treated blastemas (cluster 2). **d** Image of the tail of 4 DPF embryos injected with Cas9 or Cas9 and gRNAs against *gfpt1* and *gfpt2* (*gfpt*^kd^), set 1. Scale bar = 200 µm. **e** Quantification of tail area of 4 DPF embryos injected with Cas9 or Cas9 and gRNAs against *gfpt1* and *gfpt2* (*gfpt*^kd^), set 1. Mean and SEM are shown. *N* = 14 embryos for control and 14 embryos for *gfpt*^kd^. Statistics were determined an unpaired two-tailed *t*-test. **f** Image of 7 DPF embryo tails, 96 HPA, injected with Cas9 or Cas9 and gRNAs against *gfpt1* and *gfpt2* (*gfpt*^kd^), set 1. Scale bar = 200 µm. **g** Quantification of tail area of 7 DPF embryos, 96 HPA, injected with Cas9 or Cas9 and gRNAs against *gfpt1* and *gfpt2* (*gfpt*^kd^), set 1. Mean and SEM are shown. *N* = 20 embryos for control and 16 embryos for *gfpt*^kd^. Statistics were determined an unpaired two-tailed *t*-test. **h** Cartoon depicting glutamine amidotransferase (GATase) 6 and sugar isomerase (SIS) domains of GFPT1. TAA indicates a premature stop codon introduced by a 20 bp deletion in *gfpt1*. **i** Quantification of tail area of wild-type or gfpt1^−/−^ 7 DPF embryos, 96 HPA, injected with Cas9 or Cas9 and gRNAs against *gfpt2*. Mean and SEM are shown. Mean and SEM are shown. *N* = 18, 19, 20, and 11 embryos for wild-type control, wild-type *gfpt2*^kd^, *gfpt1*^*−*/−^, and gfpt1^−/−^; gfpt2^kd^ groups. Statistics were determined with ordinary one-way ANOVA. **j** Quantification of tail area of 96 HPA wild-type or *gfpt1*^*−/−*^ embryos with and without 10 mM 2-DG treatment. Mean and SEM are shown. *N* = 20–21 embryos for all conditions. Statistics were determined with two-way ANOVA. **k** Quantification of tail area of 4 DPF wild-type or *gfpt1*^−/−^ embryos treated with 10 mM 2-DG. Mean and SEM are shown. *N* = 23 and wild-type embryos and 15 for *gfpt1*^−/−^ embryos. Statistics were determined with an unpaired two-tailed *t*-test. **l** Quantification of tail area of 96 HPA embryos treated with DMSO or Tunicamycin. Drug was added to embryo media 2 h prior to amputations and washed out 24 HPA. Mean and SEM are shown. *N* = 19 for all conditions. Statistics were determined with an unpaired two-tailed *t*-test.

Although our data indicated that metabolic reprogramming was required for normal blastema formation, it did not determine which metabolic pathway blocked by 2-DG served as the trigger for regeneration. 2-DG inhibits hexokinase and phosphoglucoisomerase upstream of the glycolysis and HBP branchpoint, while the PPP branchpoint is downstream of hexokinase but upstream of phosphoglucoisomerase (Fig. 6a). 2-DG can also inhibit N-linked glycosylation through preventing mannose incorporation into the N-glycan core oligosaccharide, the synthesis of which also requires UDP-GlcNAc from the HBP. GO analyses of genes upregulated by 2-DG in the blastema indicated induction of the uncoupled protein response (UPR), suggesting disruption of N-linked glycosylation (Supplementary Data 5). One of the genes upregulated in the blastema of 2-DG treated samples was *gfpt1*, the gene encoding the rate-limiting enzyme of the HBP, a known compensatory effect of glucose starvation and UPR induction (Fig. 6b, c)^43,44^. A second gene in the HBP, *gnpnat1*, was also upregulated following 2-DG treatment, which together with *gfpt1* could promote flux of metabolites from carbohydrate, amino acid, and lipid metabolism into the HBP (Fig. 6c). To understand if disruption of glycosylation through inhibition of the HBP would phenocopy 2-DG, we injected active guide RNAs (gRNAs) targeting paralogs *gfpt1* and *gfpt2* along with Cas9 into zebrafish embryos (Supplementary Fig. 6a, b, Supplementary Data 6). Similar to 2-DG-treated embryos, *gfpt1* /*gfpt2* knockdown mutants showed normal tail development but failed to regenerate their tails (Fig. 6d, e, f, g). A second, nonoverlapping set of guides resulted in an identical phenotype, indicating that this was specific to *gfpt1* /*gfpt2* double mutants and not due to an off-target effect (Supplementary Fig. 6a–d). Unlike disruption of the HBP, neither stable mutations in the gene *pgk1*, which blocks glycolysis specifically, nor the rate-limiting enzyme of the PPP, *g6pd*, had any effects on regeneration (Supplementary Fig. 6e–h). Together our results indicate that glucose flux shunted to the HBP, rather than glycolysis or the PPP, is required for tail regeneration. In order to further evaluate the relationship between 2-DG and the HBP, we isolated a stable 20 bp deletion in *gfpt1* that results in a premature stop codon in the glutamine amidotransferase (GATase) 6 domain (Fig. 6h). While these mutants showed only a mild tail regeneration phenotype on their own, regeneration was essentially eliminated by injection of gRNAs against *gfpt2* or the addition of 10 mM 2-DG, a low concentration that does not affect regeneration in wild-type animals or the development of either wild-type or *gfpt1* mutant animals (Fig. 6i, j, k). This hypersensitivity of *gfpt1* mutants to 2-DG further indicates that glucose flux through the HBP is required for regeneration. Glucose derived UDP-GlcNAc from the HBP is required for many types of glycosylation. To evaluate if N-linked glycosylation or O-GlcNAcylation were involved in regeneration, we treated larvae with tunicamycin, which prevents transfer of UDP-GlcNAc to the N-glycan core oligosaccharide, or OMSI-1, a OGT inhibitor^45,46^. Similar to 100 mM 2-DG treatment, *gfpt1*/*gfpt2* knockdown, and *gfpt1* mutants treated with 10 mM 2-DG, treatment with tunicamycin strongly inhibited tail regeneration while OSMI-1 had no effect (Fig. 6l, Supplementary Fig. 6i). Together, our data indicate that metabolic reprogramming to promote glucose flux through the HBP for N-linked glycosylation is essential for TGF − β signaling and tail regeneration.

## Discussion

Despite work done to show that blastema arises from dedifferentiation of existing “fate committed” cells^47,48^, many questions remain about this process. We have shown that in the embryonic zebrafish tail, normal blastema formation requires a metabolic shift specifically to stimulate N-linked glycosylation through the HBP. This shift promotes TGF-β signaling, resulting in formation of the blastemal mesenchyme.

Although the Warburg effect has been studied for almost a century, there remains much debate as to its purpose. Initially thought to be utilized by rapidly proliferating cells, it is becoming clear that it is involved in other processes, and rapidly proliferating cells do not have to rely on glycolysis. With respect to regeneration, recent analyses of regenerating digit-tips in mice suggest that relatively few dividing cells are necessary to carry out regeneration once the blastema is formed, suggesting metabolic shifts may be related to processes other than cell division, at least in regeneration^40^. Moreover, glycolysis and its branched pathways have been shown to control cell differentiation or dedifferentiation independently of proliferation^11,49^. One pathway associated with the Warburg effect that is becoming more intensely studied in cancer cells is the HBP. Alterations to glycosylation are prevalent during cancer progression^50^, and upregulation of the HBP is crucial to these changes in glycosylation patterns^51^. Additionally, the HBP has been shown to be involved in mediating the development of mesenchymal cells associated with cancer progression^52–54^. We show that a Warburg-like physiology is associated with the HBP and N-linked glycosylation in the regenerating tail. Post amputation there is an increase of uptake of the glucose analog 2-NBDG, indicating a requirement for additional glucose during regeneration. Additionally, work from another group has shown increased lactate production during tail regeneration, indicating that glucose metabolism is uncoupled from OxPhos during this process^34^. Addition of 2-DG to embryo medium completely blocks regeneration without affecting early development, further indicating the shift to glucose metabolism is specific to regeneration. ScRNA-seq analyses showed that lack of regeneration with 2-DG treatment was due to failure to form a mesenchymal blastema, instead forming an aberrant, nonproliferative blastema. While mutations in key enzymes involved in glycolysis or the PPP had no effect on regeneration, mutating the isozymes Glutamine-Fructose-6-Phosphate Transaminase 1 and 2, the redundant, rate liming enzymes of the HBP, completely blocked regeneration. Additionally, *gfpt1* mutants were hypersensitive to 2-DG, further indicating that the specific effect of 2-DG on regeneration was through perturbation of the HBP. Similar inhibition of regeneration was obtained with tunicamycin treatment, a well-described inhibitor of N-linked glycosylation. Therefore, our results indicate that during tail regeneration, a metabolic shift takes place to promote glucose flux through the HBP and N-linked glycosylation. This supports previous findings indicating development of mesenchyme from differentiated cells results in an active secretory pathway, which requires increased flux through the HBP to maintain N-linked glycosylation and to avoid an unfolded protein response^55^. Additionally, receptor signaling of pathways involved the development of mesenchyme, such as TGF − β and EGF, are regulated by flux through the HBP^56^.

Glucose metabolism, the HBP, N-linked glycosylation, and ER homeostasis are intricately linked. Glucose deprivation can induce both the UPR and GFPT1 expression triggered by the loss of N-linked glycosylation^43,57^. Inhibition of the HBP results in decreased N-linked glycosylation and induction of the UPR^58^. Reciprocally, the UPR can induce GFPT1 expression^43,59^. And clinically, mutations in *gfpt1* present like other mutations in genes involved in N-linked glycosylation^60^. In our scRNA-seq dataset the upregulation of *gfpt1* and several genes involved in the UPR in the blastema following 2-DG treatment suggests that 2-DG inhibits N-linked glycosylation, which prevents normal blastema formation. 2-DG can perturb the glucose-HBP-glycosylation axis by (a) acting as low glucose mimetic thus preventing the synthesis of fructose-6-P and (b) directly inhibiting N-linked glycosylation by preventing mannose incorporation into Dol-PP-GlcNAc2. Interestingly, tunicamycin affects early development while *gfpt1*;*gfpt2* mutations and 2-DG treatment do not. Tunicamycin indiscriminately prevents GlcNAc incorporation into the N-glycan core oligosaccharide. However, as a glucose analog, 2-DG will exert a greater effect on tissues taking up high amounts of glucose. UDP-GlcNAc can be synthesized through a salvage pathway downstream of *gpft1/2*, which may explain how *gfpt1*/*gfpt2* mutants, although not viable beyond 10 DPF, get through early development but cannot compensate when there is an increased demand for secretory pathway activity. Together this suggests that while N-linked glycosylation is important for both early development and regeneration, there is an increased requirement for glycosylation substrates during regeneration. Yet, despite this apparent overall increase, how N-glycans are modified can have opposite effects on regeneration. As this work demonstrates, early blocks in N-glycan synthesis in the ER result in failure to develop a functional blastema. However, previous work from our lab has shown that a mutation in *mgat5* or swainosine treatment, which affect N-glycan branching in the Golgi complex, result in enhanced regeneration through modulation of TGF-β signaling^61^. Therefore, regenerative outcome is dependent on the nature of N-linked glycan modifications. Despite the clear effect of N-linked glycosylation on regeneration, we cannot rule out other forms of glycosylation or glycosoaminoglycans being involved in regeneration in addition to affecting TGF-β signaling. For example, hyaluronic acid, which requires substrate from the HBP, has also been shown to be involved in fin regeneration.

The Warburg effect is characterized by increased glucose uptake and lactate production. Although glucose flux through the HBP only accounts for an estimated 2–5% of glucose that enters the glycolytic pathway, glucose availability has a strong influence on UDP-GlcNAc synthesis and therefore it may be necessary to increase glucose uptake during regeneration to ensure adequate HBP substrate^62,63^. However, it is likely that under normal circumstances, the majority of glucose still proceeds through glycolysis. Nevertheless, blocking all glycolytic ATP with *pgk1* mutants has no regenerative phenotype despite this deletion being homozygous lethal at approximately 10 DPF. This demonstrates that ATP production from glycolysis is neither required for regeneration, nor is downstream lactic acid secretion. While glucose flux through the HBP is obligate for regeneration, there may be metabolic plasticity for ATP production as seen in tumor cells^64^. Consistent with metabolic plasticity, impairing mitochondrial function or glycolysis has little to no effect on regeneration, indicating that the source of ATP during regeneration is inconsequential.

TGF-β signaling is regulated by metabolic reprogramming and is essential for tail regeneration. The activin genes *inhbaa* and *inhbb* are initially transcriptionally repressed by 2-DG at 24 HPA in the notochord bead, but *tgfb1* is not repressed until later in the regenerative process. Accordingly, by assaying phospho-SMAD2 activity, we did not observe a decrease in overall TGF-β signaling with 2-DG treatment until 48 HPA in the blastema, when both the activin genes and *tgfb1* were repressed. This potentially explains why an aberrant, nonproliferative blastema, rather than no blastema, forms when glycosylation is inhibited. We hypothesize that initial TGF-β signaling through Tgfb1 begins the dedifferentiation process and is independent of a metabolic shift to promote glycosylation, but the shift is necessary to maintain a sustained TGF-β signal which initiates blastemal mesenchyme formation, promotes cell proliferation, and makes the blastema competent for coordinated regeneration. In support of this hypothesis *snai1a*, which is essential for mesenchyme development from differentiated cells, is suppressed by both 2-DG or inhibition of TGF-β signaling. Metabolic reprogramming, including shunting to the HBP, has been proposed to take place in the blastema during both lizard tail regeneration^5,6^, and regulation of signaling by N-linked glycosylation has been demonstrated to take place in the blastema during axolotl limb regeneration. Additionally, TGF-β is required for blastema formation in lizard tail regeneration, zebrafish fin regeneration, axolotl limb regeneration, and *xenopus* tail regeneration^42,65–67^. Interestingly, in adult zebrafish and gecko tails, activin appears to be the primary TGF-β ligand, while in our dataset, both activin and TGF-β1 are inhibited by 2-DG treatment^42,65^. It is possible that activin induced by metabolic reprogramming during regeneration is a conserved mechanism in regenerative species.

A previous report demonstrated that notochord streaming was triggered through ROS driven hedgehog signaling and was necessary for regeneration^19^. ROS generation and substrate availability for glycosylation both depend on regulation of cellular metabolism, and reflect the complex, interdependent nature of cellular responses to injury. Additionally, links between ROS and alterations to glycans have been suggested^68^. However, our data, in contrast to ROS, show no evidence that glycosylation regulates sonic hedgehog ligands in the notochord bead or blastema. This suggests that ROS and glucose flux through the HBP are activating distinct, parallel pathways, or alternatively ROS and sonic hedgehog act upstream of the glycolytic shift and the subsequent TGF-β signaling. Further research will be required to tease apart how metabolic changes trigger the various signaling cascades required for regeneration.

With respect to mammals, the MRL strain of mouse, which has superior regenerative capacity compared to wild-type mice, has higher levels of aerobic glycolysis than normal mice, potentially preserving some modest ability to trigger blastema formation, at least under limited circumstances^69^. Additionally, several studies indicate that a glycolytic shift is required for dedifferentiation of mammalian cells in vitro^15,70,71^. Although it remains to be determined whether this switch to glycolysis is, at least in part, to maintain intermediates for pathways such as the HBP, pluripotent stem cells have been shown to have a distinct N-glycan profile and the HBP has been implicated in maintaining pluripotency^72,73^. Notably, the HBP has been shown to drive the Warburg effect^51,74^. Therefore, our findings may contribute to our understanding of regeneration and wound healing on a broader scale.

In addition to tissue regeneration and stem cell biology, blastema formation shows several parallels to cancer biology, including a shift to glucose-dependent metabolism, development of mesenchyme from previously differentiation cells, and cell migration. However, unlike tumor progression, regeneration is spatially and temporally coordinated, allowing us to control variables and obtain insight into critical but transient gene expression within subpopulations of cells. By using scRNA-seq, we were able to capture a small population of blastema cells that lacked mesenchymal markers, possibly indicating that these cells were on their way to becoming the mesenchymal blastema. One of the most significantly enriched genes in this population was transgelin (*tagln*) (Supplementary Data 3). Transgelin is an actin binding protein expressed in smooth muscle, fibroblasts, and the umbilical cord mesenchymal stem cells. Furthermore, its expression has been detected in cancer stem cells and has been implicated in their migration and invasion^75^. Paradoxically, trangelin has been described as both promoting and inhibiting bladder cancer^76,77^. The paradox is potentially resolved if timing and transient transgelin expression is important in the progression of bladder cancer as it may be in the formation of a blastema. Due to many observed similarities between wound healing and cancer, cancer has been described as a wound that does not heal^78^. Moreover, multiple studies showing that both wound healing (or regeneration) and cancer can have similar transcriptional profiles^79,80^. Therefore this work may provide insight into the cellular processes that drive tumor progression as well as tissue regeneration^6,65,66,81^.

## Methods

### Zebrafish husbandry

Adult zebrafish were maintained at 28 °C with a 14 h light/10 h dark cycle. Embryos collected from crosses were staged as previously described^82^. All animal experiments were performed in compliance with NIH guidelines for ethical animal handling and research was approved by the Animal Care and Use Committee of the National Human Genome Research Institute (Protocol G-01-3). TAB-5 were used for all experiments unless otherwise noted. Sex could not be determined at the ages of the animals used but is assumed to be evenly distributed between male and female in the offspring.

### Plasmid synthesis

Plasmids were generated with multisite gateway technology (Invitrogen). To obtain Tg(*actb2*:MLS-EGFP), we generated a middle entry clone with the human COXVIII mitochondrial localization sequence (MLS) fused upstream of GFP^36^, which we recombined with p5E β-actin2, p3E-polyA, and pDestTol2pA2 from the tol2 kit^83^. To generate Tg(*actb2*:MLS-roGFP2), a middle entry clone containing the zebrafish COXVIII MLS and p3E-roGFP2 were recombined with p5E β-actin2 and pDestTol2pA2 from the tol2 kit.

### Generation of transgenic zebrafish

To synthesize *tol2* mRNA, pCS2FA-transposase was linearized using NotI (New England Biolabs), then purified using a PCR purification kit (Qiagen). mRNA was synthesized with mMessage mMachine SP6 Kit (Ambion) and purified with RNA clean and concentrator kit (Zymo Research). 1-cell stage TAB-5 embryos were injected with 50 pg *tol2* mRNA and 25 pg plasmid. Injected embryos expressing mosaic GFP were grown to adults and screened for germline transmission. Mitochondrial localization of GFP and roGFP in our transgenic lines was verified by soaking 3 DPF embryos in 50 nM mitotracker red CMXRos (ThermoFisher) for 1 h and analyzing caudal fin-fold epithelium for co-fluorescence.

### Generation of mutant zebrafish

Mutant zebrafish were generated and screened according to a previously described protocol^84^. In brief, 50 pg of gRNAs and 1 µl of Cas9 protein (New England Biolabs) were injected into embryos during the 1-cell stage. Embryos were grown to adults and outcrossed to screen for transmission of out-of-frame alleles.

### Pharmacological studies

To inhibit glycolysis, 2-Deoxy-D-glucose (Sigma) powder was added to holtfreter’s buffer to a final concentration of 100 mM. Glucose (Sigma) was used as a control. To inhibit mitochondrial protein import, a 50 mM stock of Mitoblock-6 in DMSO (Calbiochem) was diluted into holtfreter’s buffer and 1% DMS0 to a final concentration of 2.5 µM. To inhibit TGF-β signaling, a 20 mM stock of SB431542 (Sigma) in DMSO was diluted into holtfreter’s solution to a final concentration of 50 µM. To inhibit N-linked glycosylation, a 5 mg/ml stock of tunicamycin (Cayman Chemical) was diluted into holtfreter’s solution to a final concentration of 1 µg/ml. To inhibit O-GlcNAcylation, a 20 mM stock of OSMI-1 (Cayman Chemical) was diluted in holtfreter’s buffer to a final concentration of 50 µM. For development assays, treatments were initiated at 1 DPF following dechorionation of embryos with 1 mg/ml pronase (Sigma) for 15 min and continued until 4 DPF when embryos were analyzed. For regeneration assays, treatments were initiated 2 h prior to amputation and continued throughout the duration of the experiment unless otherwise noted. Inhibitors were washed out and replaced every 24 h for all experiments unless otherwise noted.

### Tail area quantification

Tail amputations were performed as previously described^85^. All amputations were performed at 3 DPF. Relative tail size was quantified using the region of interest method in FIJI. Tails were measured from the posterior end of the notochord to the posterior end of the caudal fin-fold. Area was normalized to 100% using wild type as the reference.

### In situ hybridization

In situ hybridization was performed as previously described^86^. Primers used to amplify template cDNA were *snai1a*: For-5’ GCGGGTCCAAATGGACGTAAACC, Rev-5’ TGGCGGCAGTAACTGCAGC; *msx3*: For-5’ AGTGCGCTCTGCTTGGAGAGC, Rev-5’ GTGCTTCAGTCTGTTAAGACAAATAATACATTCCGTAGG. A T7 promoter was included on the reverse primers. Probes were synthesized with T7 polymerase and DIG labeling mix (Roche 10881767001, 11277073910).

### RNA fluorescence in situ hybridization (RNA-FISH)

HCR V3.0 probes were purchased from Molecular Instruments. RNA-FISH was performed according to manufacturer’s protocol for whole mount zebrafish embryos.

### Immunofluorescence

Embryos were fixed in 4% paraformaldehyde (PFA) for 45 min at room temperature, washed with PBST, and incubated in 1 mg/mL collagenase for 2 h. Embryos were washed with PBST and frozen in acetone at −20 °C for 10 min. Embryos were washed in PBST and incubated in 5% goat serum (Thermofisher NC9270494) in BDP (PBS/0.1% BSA/1% DMSO) for 1 h. Embryos were incubated with anti-phospho-SMAD2 primary antibody (Cell Signaling E8F3R) diluted 1:800 in BDP or anti-phospho-histone H3 (Ser10) primary antibody (Cell Signaling D2C8) diluted 1:200 in BDP overnight at 4 °C, followed by incubation with secondary antibody (Alexa Fluor 488 A-11008) diluted in 1:500 BDP for 1 h at RT. Nuclei were stained by incubating with Hoechst 33342 (Thermofisher) diluted 1:2000 in PBST for 20 min at RT.

### 2-NBDG uptake

Embryos were incubated in 500 µM 2-NBDG (Cayman) in Holtfreter’s buffer for 2 h. Embryos were then anesthetized with MS-222 and embedded in 0.8% agarose for microscopy.

### FITC-dextran permeability assay

FITC-dextran, average mol wt 3000–5000 (Sigma) was dissolved in Holtfreter’s buffer to a concentration of 1 mg/ml. Uninjured or amputated embryos were incubated in the solution for 30 min, then anesthetized with MS-222 and embedded in 0.8% agarose for microscopy and imaged using FITC filters.

### TUNEL staining

Apoptosis was analyzed using in Situ Cell Death Detection Kit, TMR red (Roche). Embryos were fixed in 4% PFA in 1× PBS for 24 h at 4 °C. Embryos were then washed twice in 1× PBS for 5 min each. Embryos were dehydrated in 50% methanol for 5 min, then 100% methanol for 5 min. Samples were stored in −20 °C overnight. The following day, embryos were rehydrated with 75% methanol in PBST (PBS, 1% Tween) for 5 min, 50% methanol in PBST 5 min, 25% methanol in PBST 5 min, 100% PBST 2× for 5 min. Embryos were exposed to a proteinase K wash for 10 min at 37 °C, the fixed again in 4% PFA for 20 min. Embryos were then washed for 1 hr (3× for 20 mins) with PBSTX (PBS, 1% Triton-X). TUNEL reaction mixture was prepared and added to each samples. Samples were incubated for 60 min at 37 °C covered in foil. Samples were then washed 2× with PBSTX for 5 min each time and analyzed by confocal microscopy.

### Microscopy

Embryos were anesthetized with MS-222 (Sigma) and embedded in 0.8% agarose (Fisher) for imaging. Epifluorescence images were acquired with an Axiovert 200 M (Zeiss) and an Orca-ER camera (Hamamatsu). Bright field images were acquired with an MZ16F L3 (Leica) and Axio Cam HR (Zeiss). Confocal images were acquired with an LSM 880 (Zeiss). Images were analyzed in FIJI^87,88^. For time-lapse microscopy of Tg(*col9a2*:mCherry; *col8a1a*:GFP) embryos, embryos were anesthetized in holtfreter’s buffer (59 mM NaCl, 0.88 mM CaCl_2_·2H_2_0, 0.67 mM KCl, 5 mM HEPES) containing 600 µM MS-222 (Sigma) and embedded in 1% low-melt agarose (NuSieve GTG) containing MS-222 on glass-bottomed microwell dishes (MatTek 35 mm). Imaging was performed using a Nikon W1 spinning disc confocal microscope. After acquisition, image sequences were bleach corrected using histogram normalization^89^, and channels merged in FIJI.

### Mitochondrial volume quantification

Segmentation was performed with the Allen Institute for Cell Science Segmenter Python library^90^ using the Tom20 workflow. A vesselness sigma and cutoff of 1 and 0.025, respectively, were used. Following segmentation, small objects of less than 50 voxels and large objects of more than 10,000 voxels were filtered out to reduce segmentation artifacts. The remaining objects were counted, and their areas determined using the scikit-image measure module^91^.

### P-Smad2 intensity quantification

Nuclei from the Hoechst channel were identified by the “Find Edges” and “Bernsen Automatic Thresholding” functions in FIJI. Quantification of particle intensity was then redirected to the P-Smad2 IF channel.

### Single-cell RNA seq

Tails were amputated at the posterior end of the pigment gap and collected as uninjured samples or embryos were placed back into media to allow tail regeneration. Regenerating tails were collected by performing a second amputation at the anterior end of the pigment gap at 24 or 48 HPA. Eighty tails were collected per sample. Amputated tails and tail regenerates were dissociated for single-cell RNA-seq analysis using a previously described protocol with 1% BSA used in place of FBS^92^. Following dissociation to single cells, cell viability and concentration was analyzed using a Luna cell counter. Samples consistently had greater than 90% cell viability. Gel Bead-in-Emulsions were prepared by loading 10,000 cells per sample in 46.6 µL DMEM + 1% BSA onto a Chromium Chip B (10× Genomics 1000073) and run using the Chromium Controller (10× Genomics). cDNA libraries were generated with Chromium Single Cell 3’ GEM, Library and Gel Bead Kit V3 (10× Genomics). Libraries were sequenced using the NextSeq 500/550 High Output Kit v2.5 (Illumina) on an Illumina NextSeq 550. Reads were aligned to the zebrafish genome ref. ^11^ and feature-barcode matrices were generated with Cell Ranger 3.0.2 (10× Genomics) using default parameters. Valid barcode reads in all samples were >90%. Reads aligned to zebrafish genome were >80% in all samples, while anti-sense reads were <1% in all samples. All key metrics were within the expected range and did not trigger any errors or warnings in the Cell Ranger web summaries. Three aggregate files were made using Cell Ranger to create a single matrix of all included samples: (1) Uninjured, 24 HPA, and 48 HPA control samples; (2) 24 HPA and 48 HPA control and 2-DG treated samples; (3) uninjured control and uninjured 2-DG treated samples. T-SNE plots, violin plots, and differential gene expression lists were generated with Loupe Viewer version 5.0 (10× Genomics) using default settings.

### Gene list analyses

Genes list analyses were performed using the Panther Classification System 14.1 (www.pantherdb.org)^93^. Statistical Overrepresentation tests were performed with Fisher’s exact test and corrected with False Discovery Rate.

### Heatmap generation

Heatmaps were generated using Heatmapper (http://www2.heatmapper.ca/).

### Statistical analyses

Unpaired two-tailed *t*-tests, ordinary one-way ANOVA, and two-way ANOVA analyses were performed in Graphpad Prism 9. All experiments were repeated a minimum of two times for statistical analyses. Embryos were measured once each. Differential gene expression from scRNA-seq data was determined using Loupe Viewer software version 5.0 (10× genomics), which employs a negative binomial exact test and Benjamini–Hochberg correction for multiple tests^94^.

### Reporting summary

Further information on research design is available in the Nature Research Reporting Summary linked to this article.

## Supplementary information

Supplementary Movie 1

Supplementary Information

Supplementary Data 1

Supplementary Data 2

Supplementary Data 3

Supplementary Data 4

Supplementary Data 5

Supplementary data 6

Reporting Summary

## Data Availability

All data were deposited to GEO under accession GSE145497. There are no restrictions on data availability.
