# Supplementary material for: The Warburg effect is necessary to promote glycosylation in the blastema during zebrafish tail regeneration
Source: NPJ Regen Med. 2021 Sep 13;6:55. doi: 10.1038/s41536-021-00163-x (PMC8437957; doi:10.1038/s41536-021-00163-x)
Supplement: Supplementary file 2 — Supplementary Information [file 41536_2021_163_MOESM2_ESM.pdf]

Supplementary Figure 1

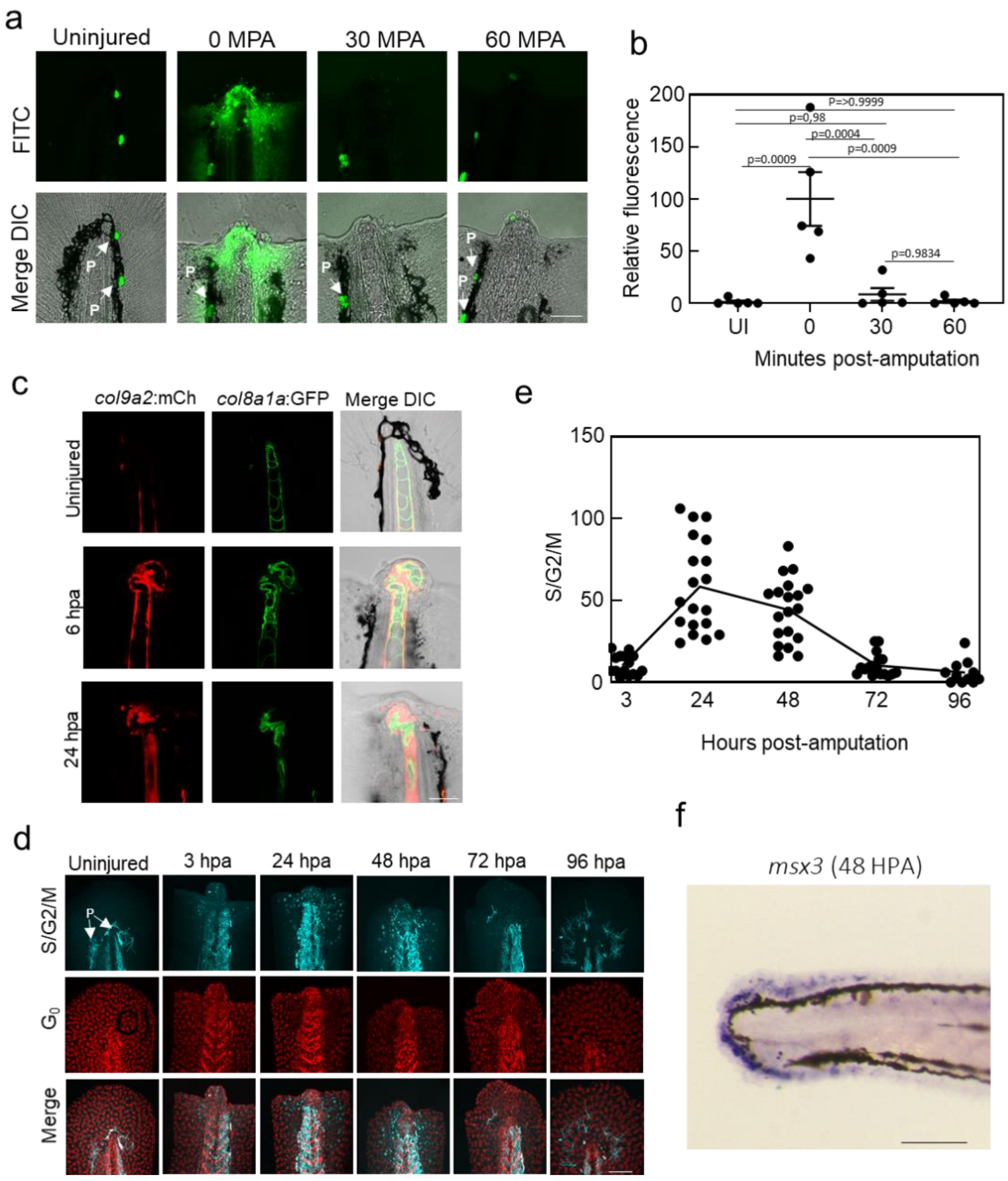

Supplementary Figure 1: Processes involved in early tail regeneration

a) Confocal images of uninjured or amputated 3 DPF embryos pulsed with 1 mg / ml FITC-dextran for 30 minutes. Images are sum z-stack projections of 1 µM slices through the entire embryo. P indicates autofluorescent pigment cells. Scale bar = 50 µm. b) Quantification of fluorescence in 3 DPF embryos pulse with 1 mg / ml FITC-dextran at indicated timepoints. Mean and SEM are shown. N=5 embryos for all samples. Statistics determined by ordinary one-way ANOVA. c) Confocal images of uninjured, 6 HPA, or 24 HPA Tg(*col9a2*:mCherry; *col8a1a*:GFP) embryos. Scale bar = 50 µM. Images are single 1

µM z-slice through the notochord. d) Tail images of the Dual Fucci transgenic fish following tail amputation at indicated timepoints in hours post amputation. Cells in S, G2, and M phase are depicted by cyan fluorescence in the nucleus. Cells in G0/1 phase are depicted in red. P indicates auto-fluorescent pigment cells which are not expressing Tg(*ubb*:Cerulean-gmnn). Images are sum z-stack projections of 1 µM slices through the entire embryo. Scale bar = 100 µm. e) Quantification of cell proliferation in Dual Fucci transgenic fish and tail area during regeneration. N=20 embryos for all timepoints. f) In situ hybridization of the blastema marker *msx3* in 5 DPF embryos at 48 HPA. Embryos were not treated with 1-phenyl 2-thiourea to help demonstrate the mesenchymal expression of *msx3*. Scale bar = 100 µm.

Supplementary Figure 2

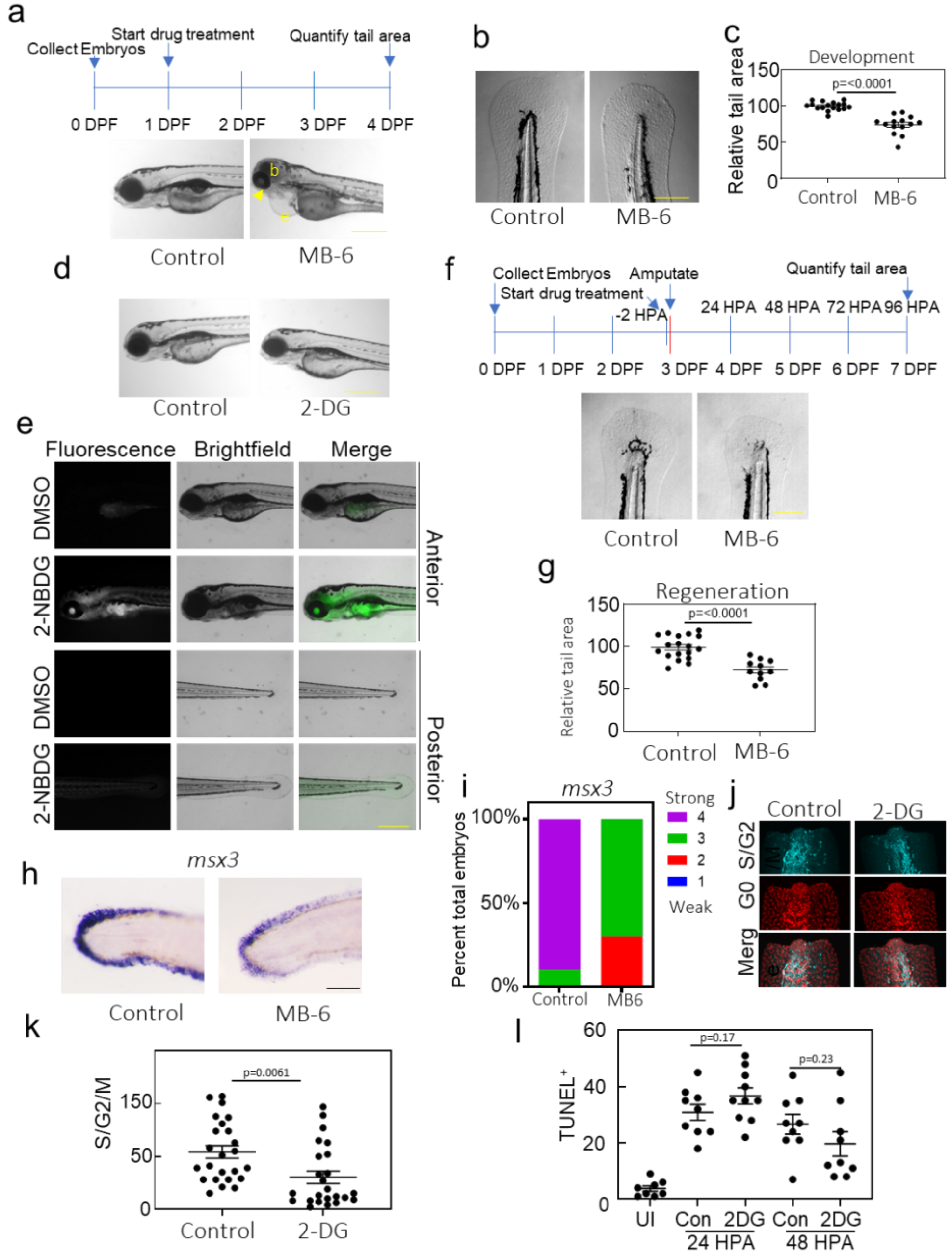

Supplementary Figure 2: Mitochondrial function but not glucose metabolism is essential for embryonic development

a) Images of 4 DPF embryos treated with 1% DMSO (control) or 2.5 µM MB-6 from 1-4 DPF. b, blood pooling in the head. e, edema. Arrow indicates underdeveloped eye. Scale bar = 500 µm. b) Images of tails of 4 DPF embryos treated with 1% DMSO (control) or 2.5 µM MB-6 from 1-4 DPF. Embryos were treated 1 DPF through the duration of the experiment. Scale bar = 200 µm. c) Quantification of tail surface area at 4 DPF of embryos treated with 1% DMSO (control) or 2.5 µM MB-6. Mean and SEM are shown. N=17 embryos for control and 14 embryos for MB-6 treatment. Statistics were determined with an unpaired two-tailed t-test. d) Images of 4 DPF embryos untreated (control) or treated with 100 mM 2-

DG from 1-4 DPF. Scale bar = 500 µm. e) Fluorescence and brightfield images of 3 DPF embryos pulsed with 500 µM 2-NBDG for 2 hrs. Scale bar = 500 µm. f) Images of tails of 7 DPF embryos, 4 days post-amputation treated with 1% DMSO (control) or 2.5 µM MB-6. Scale bar = 200 µm. g) Quantification of tail surface area at 96 HPA of embryos treated with 1% DMSO (control) or 2.5 µM MB-6. Embryos were treated 2 hours before amputation through the duration of the experiment. Mean and SEM are shown. N=18 embryos for control and 11 embryos for MB-6 treatment. Statistics were determined with an unpaired two-tailed t-test. h) In situ hybridization of *msx3* in DMSO (control) or MB-6 treated embryos at 48 HPA. Scale bar = 100 µm. i) Stacked percentage graph of *msx3* expression as determined by *in situ* hybridization. A score of 1 represents little to no staining, while a score of 4 represents strong staining. N= 10 embryos for all samples. j) Confocal images untreated and 2-DG treated Fucci fish embryo tails at 24 HPA. Images are sum z-stack projections of 1 µM slices through the entire embryo. Scale bar = 50 µm. k) Quantification of cells in S, G2, or M phase in untreated or 2-DG treated Fucci embryo tails 24 HPA. Mean and SEM are shown. N=24 embryos per condition. Statistics were determined with an unpaired two-tailed t-test. l) Quantification of TUNEL staining in tails of uninjured or 24 and 48 HPA embryos. Mean and SEM are shown. N= 8-10 embryos for all conditions. Statistics were determined with individual unpaired two-tailed t-tests between control and 2-DG embryos at 24 and 48 HPA.

Supplementary Figure 3

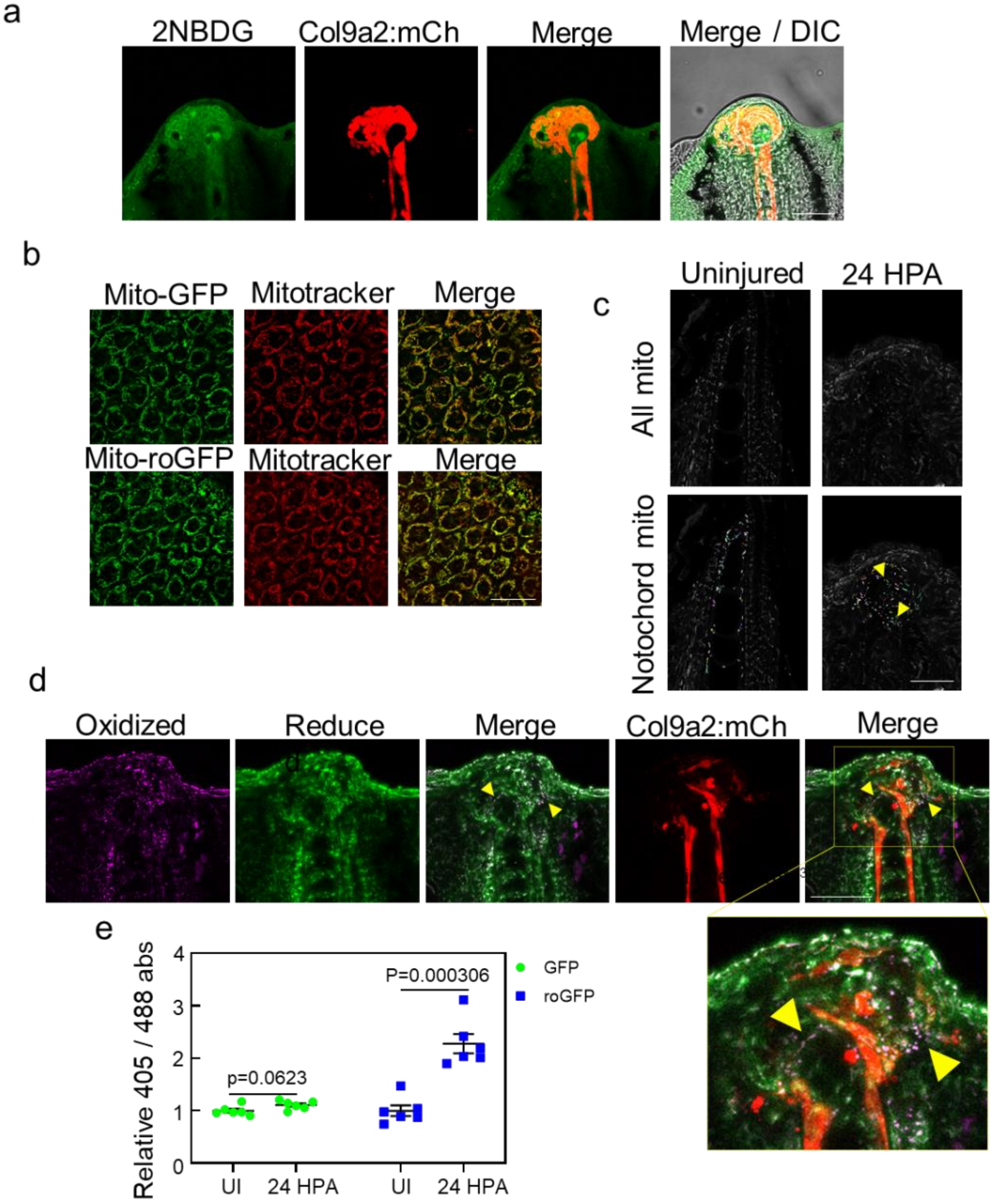

Supplementary Figure 3: Glucose uptake and mitochondrial redox in the notochord bead

a) Confocal images of Tg(*col9a2*:mCherry) embryo pulsed with 500 µM 2-NBDG for 2 hours 24 HPA. Images are a single 1 µM z-slice through the notochord bead. Scale bar = 100 µM. b) Confocal images of caudal fin-fold epithelium of Tg(*actb2*:mito-GFP) and Tg(*actb2*:mito-roGFP2) pulsed with mitotracker red for 1 hour. Images are a single 1 µM z-slice through the caudal fin-fold epithelium. Scale bar = 25 µM. For mito-GFP, the average Mander's coefficient above threshold across 3 images is 0.87 (mito-GFP) and 0.92 (mitotracker) indicating strong co-localization. For mito-roGFP, the average Mander's coefficient above threshold across 3 images is 0.89 (mito-roGFP) and 0.83 (mitotracker) indicating strong co-localization. c) Confocal images of uninjured and 24 HPA Tg(*actb2*:mito-GFP; *col9a2*:mCherry) with

and without segmenting out notochord and notochord bead mitochondria using *col9a2*:mCherry expression. Arrowhead indicates fragmented mitochondria in notochord bead. Scale bar = 100 µM. d) Confocal images of a 24 HPA Tg(*actb2*:mito-roGFP; *col9a2*:mCherry) embryo. Images are a single 1 µM z-slice through the notochord bead. Arrowheads indicate hyperoxidized mitochondria within the notochord bead. Scale bar = 100 µM. e) Quantification of relative absorbance of mitochondria targeted GFP or roGFP2 from strains Tg(*actb2*:mito-GFP) and Tg(*actb2*:mito-roGFP2) at 488 or 405 nm in the tail of uninjured embryos or the notochord bead of 24 HPA embryos. Mean and SEM are shown. N=6 embryos for each condition. Statistics were determined with individual unpaired two-tailed t-tests.

Supplementary Figure 4

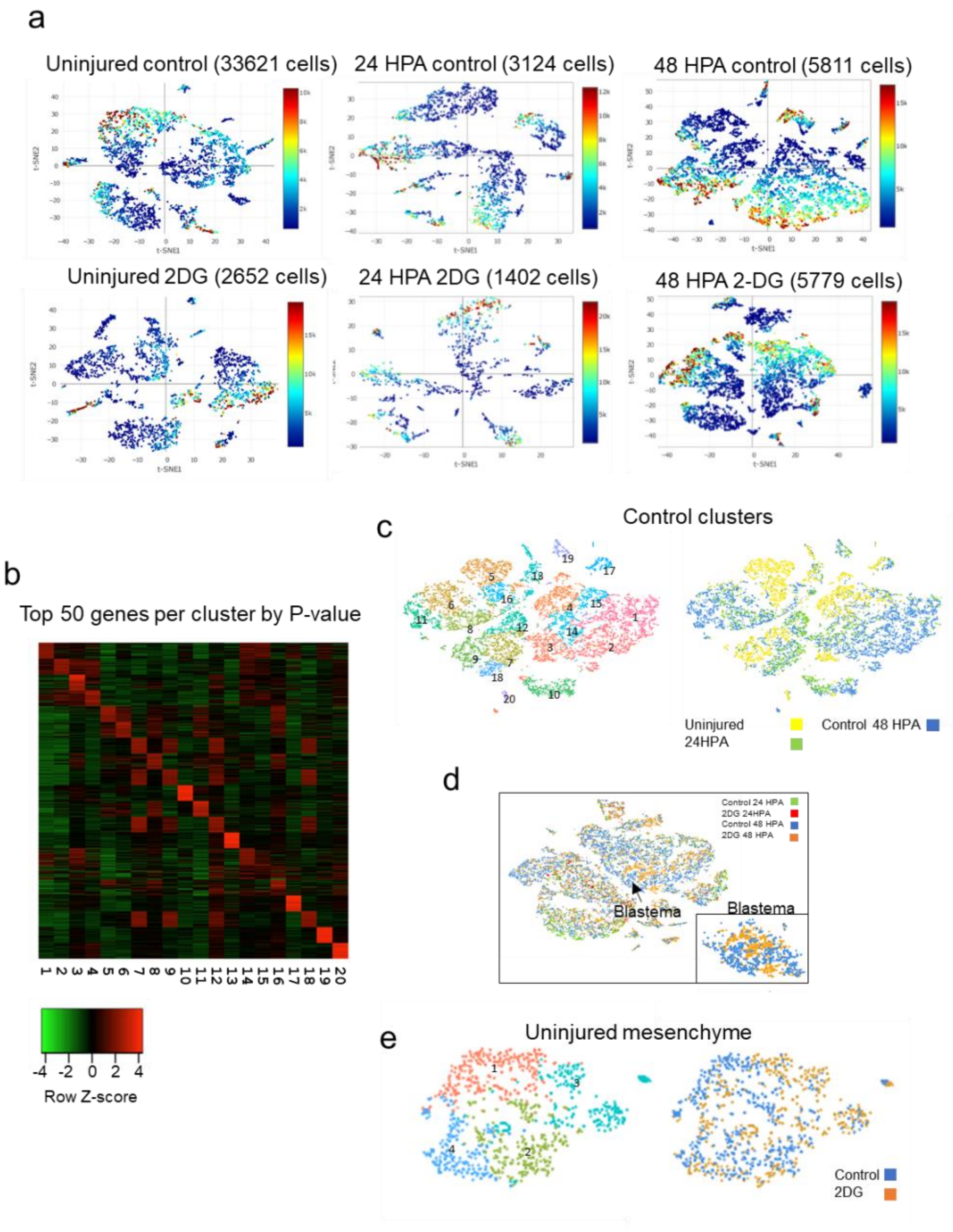

Supplementary Figure 4: scRNAseq of regenerating tail

a) t-SNE plots from each library color coded by unique molecular identifier (UMI) counts. b) Heatmap of relative gene expression of the top 50 markers for each cluster as determined by p-value. c) t-SNE plot of untreated control cells from scRNA-seq shown clusters and by library. d) t-SNE plot of from scRNA-seq of regenerating tails by library. e) Clusters generated by reclustering mesenchymal cells from uninjured samples shown by cluster number and by library.

Supplementary Figure 5

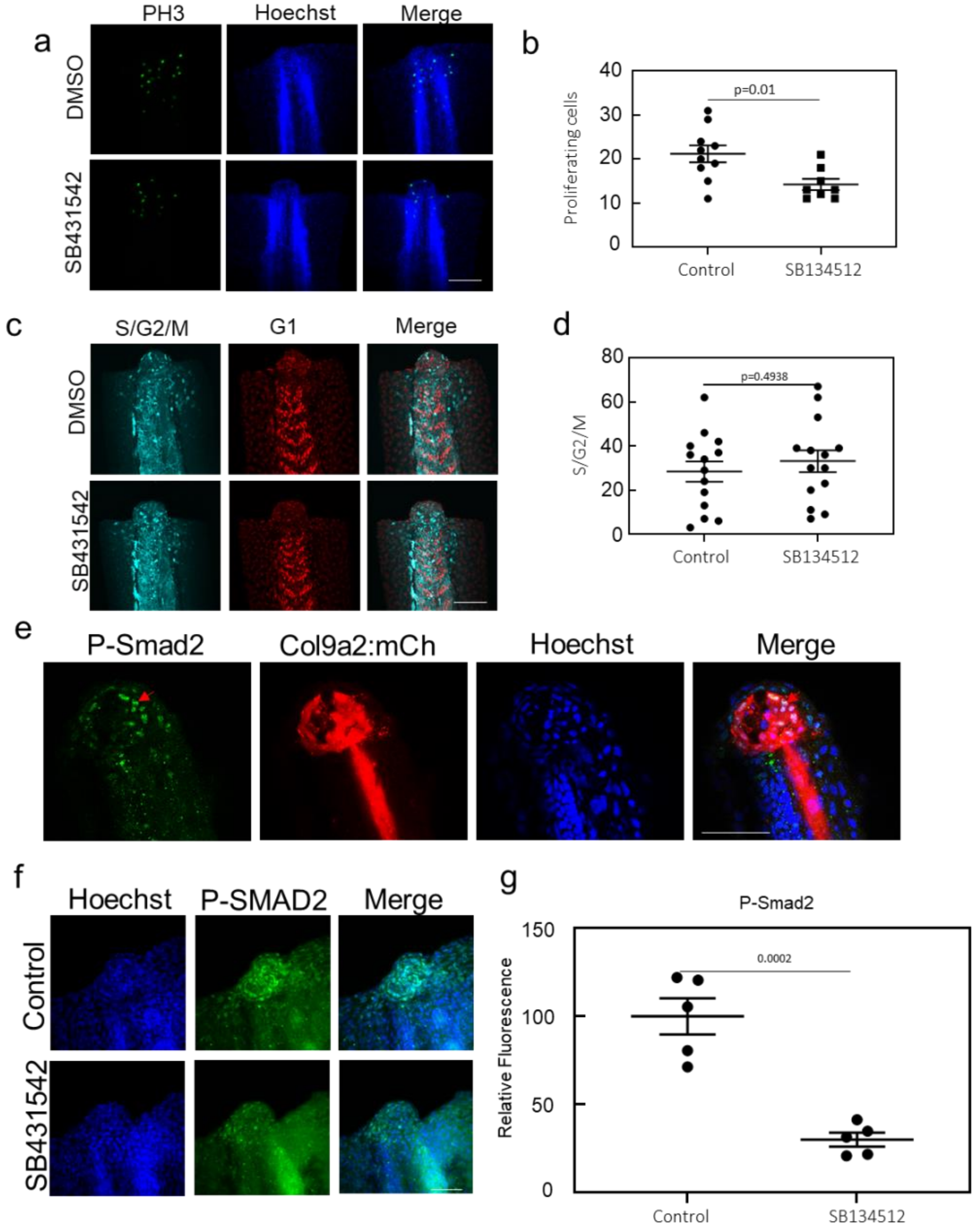

Supplementary Figure 5: SB431542 reduces TGF-β signaling

a) Confocal images pf PH3 staining in DMSO and SB431542 treated embryo tails at 24 HPA. Images are sum z-stack projections of 1 µM slices through the entire embryo. Scale bar = 50 µm. b) Quantification of proliferating cells as determined by PH3 staining. Mean and SEM are shown. N=8-10 embryos for each condition. Statistics were determined by an unpaired two-tailed t-test. c) Confocal images of DMSO or SB431542 treated Fucci fish embryo tails at 24 HPA. Images are sum z-stack projections of 1 µM slices through the entire embryo. Scale bar = 50 µm. d) Quantification of cells in S, G2, or M phase in DMSO

or SB431542 treated Fucci embryo tails at 24 HPA. Mean and SEM are shown. N= 14 embryos per condition. Statistics were determined using an unpaired two-tailed t-test. e) Confocal image of P-Smad2 immunofluorescent staining in a 24 HPA Tg(*col9a2*:mCherry) embryo. Images are a single 1 µM z-slice through the notochord bud. Scale bar = 25 µm. f) Immunofluorescent staining of P-Smad2 in control or SB431542 treated 24 HPA embryos. Images are sum z-stack projections of 1µM slices through the entire embryo. Scale bar = 50 µm. g) Quantification of P-Smad2 IF fluorescence in the notochord bead of 24 HPA control or SB431543 treated embryos. Mean and SEM are shown. N=10 embryos for all samples. Statistics were determined by an unpaired two-tailed t-test.

Supplementary Figure 6

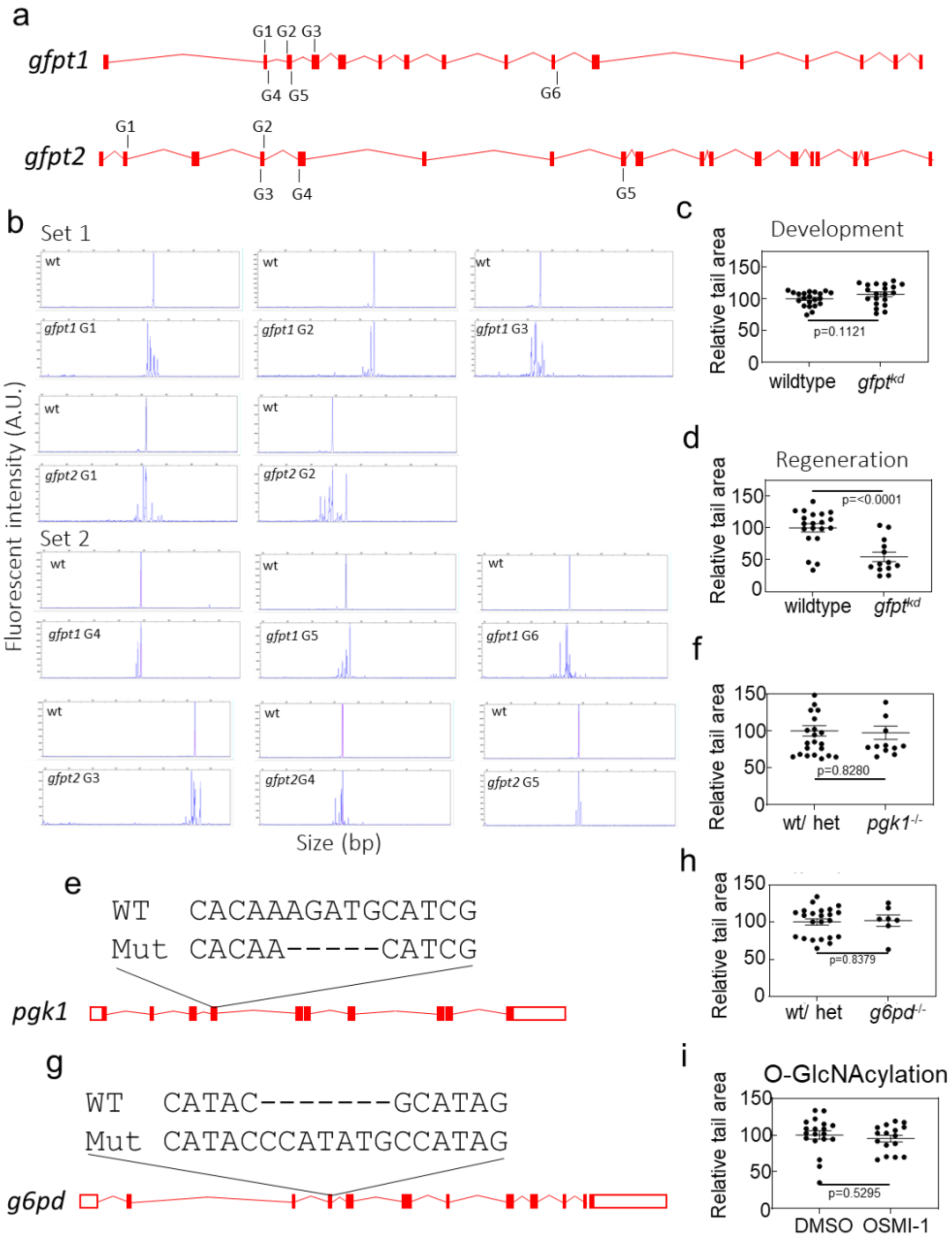

Supplementary Figure 6: CRISPR/Cas9 mediated mutation of glycolysis and branched pathway genes

a) Diagram depicting gRNA binding sites within exons of *gfpt1* and *gfpt2*. b) Fluorescent PCR traces of wild-type (wt) or *gfpt1* /*gfpt2* injected embryos. X axis is bp, Y axis is peak height. c) Quantification of tail area of 4 DPF embryos injected with Cas9 or Cas9 and gRNAs against *gfpt1* and *gfpt2* (*gfpt*^{kd}), set 2. Mean and SEM are shown. N=21 embryos for control and 20 embryos for *gfpt*^{kd}. Statistics were determined with an unpaired two-tailed t-test. d) Quantification of tail area of 7 DPF embryos, 96 HPA, injected with Cas9 or Cas9 and gRNAs against *gfpt1* and *gfpt2* (*gfpt*^{kd}), set 1. Mean and SEM are shown.

N=20 embryos for control and 13 embryos for *gfpt*^{kd}. Statistics were determined with an unpaired two-tailed t-test. e) Wild-type (WT) and mutant sequence in exon 4 of *pgk1*. f) Quantification of tail area of 7 DPF, 96 HPA mutant *pgk1* embryos or wildtype/ heterozygous siblings. Mean and SEM are shown. N=24 embryos for control and 13 embryos for *pgk1*$^{-/-}$. Statistics were determined with an unpaired two-tailed t-test. g) Wild-type (WT) and mutant sequence in exon 4 of *g6pd*. h) Quantification of tail area of 7 DPF, 96 HPA mutant *g6pd* embryos or wild-type/ heterozygous siblings. Mean and SEM are shown. N=23 embryos for control and 7 embryos for *g6pd*$^{-/-}$. Statistics were determined with an unpaired two-tailed t-test. i) Quantification of tail area of 96 HPA embryos treated with DMSO or 50 µM OSMI-1. Drug was added to embryo media 2 hours prior to amputations and treatment persisted throughout the duration of experiment. Mean and SEM are shown. N=19 embryos for control and 16 embryos for OSMI-1 treatment. Statistics were determined with an unpaired two-tailed t-test.
