# Supplementary material for: The Warburg effect is necessary to promote glycosylation in the blastema during zebrafish tail regeneration
Source: NPJ Regen Med. 2021 Sep 13;6:55. doi: 10.1038/s41536-021-00163-x (PMC8437957; doi:10.1038/s41536-021-00163-x)
Supplement: Supplementary file 9 — Reporting Summary [file 41536_2021_163_MOESM9_ESM.pdf]

nature portfolio

Corresponding author(s): *Double-blind peer review submissions: write DBPR and your manuscript number here instead of author names.*

Last updated by author(s): *YYYY-MM-DD*

Reporting Summary

Nature Portfolio wishes to improve the reproducibility of the work that we publish. This form provides structure for consistency and transparency in reporting. For further information on Nature Portfolio policies, see our Editorial Policies and the Editorial Policy Checklist.

Statistics

For all statistical analyses, confirm that the following items are present in the figure legend, table legend, main text, or Methods section.

n/a	Confirmed	
☐	☒	The exact sample size (*n*) for each experimental group/condition, given as a discrete number and unit of measurement
☐	☒	A statement on whether measurements were taken from distinct samples or whether the same sample was measured repeatedly
☐	☒	The statistical test(s) used AND whether they are one- or two-sided<br>*Only common tests should be described solely by name; describe more complex techniques in the Methods section.*
☒	☐	A description of all covariates tested
☐	☒	A description of any assumptions or corrections, such as tests of normality and adjustment for multiple comparisons
☐	☒	A full description of the statistical parameters including central tendency (e.g. means) or other basic estimates (e.g. regression coefficient) AND variation (e.g. standard deviation) or associated estimates of uncertainty (e.g. confidence intervals)
☐	☒	For null hypothesis testing, the test statistic (e.g. *F*, *t*, *r*) with confidence intervals, effect sizes, degrees of freedom and *P* value noted<br>*Give P values as exact values whenever suitable.*
☒	☐	For Bayesian analysis, information on the choice of priors and Markov chain Monte Carlo settings
☒	☐	For hierarchical and complex designs, identification of the appropriate level for tests and full reporting of outcomes
☒	☐	Estimates of effect sizes (e.g. Cohen's *d*, Pearson's *r*), indicating how they were calculated

Our web collection on statistics for biologists contains articles on many of the points above.

Software and code

Policy information about availability of computer code

Data collection	Illumina NextSeq System Suite v2.5 was used to collect cDNA sequences. Nikon NIS-Elements AR 5.20.02 64-bit was used to time-lapse imaging. Zeiss Zen black v2.3 was used for confocal imaging. Axiovision 4.8.2 was used for epifluorescence imaging.
Data analysis	FIJI was used for image analyses. Prism 9 (Graphpad) was used for statistical analyses. Cell Ranger 3.0.2 (10x Genomics) and Loupe Viewer software version 5.0 (10x genomics) were used for scRNAseq analyses. Cell Science Segmenter Python library using the Tom20 workflow and the scikit-image measure module were used to measure mitochondrial volume. Genes list analyses were performed using the Panther Classification System 14.1.

For manuscripts utilizing custom algorithms or software that are central to the research but not yet described in published literature, software must be made available to editors and reviewers. We strongly encourage code deposition in a community repository (e.g. GitHub). See the Nature Portfolio guidelines for submitting code & software for further information.

Data

Policy information about availability of data

All manuscripts must include a data availability statement. This statement should provide the following information, where applicable:

- Accession codes, unique identifiers, or web links for publicly available datasets
- A description of any restrictions on data availability
- For clinical datasets or third party data, please ensure that the statement adheres to our policy

All scRNAseq data was deposited to GEO under accession GSE145497

Field-specific reporting

Please select the one below that is the best fit for your research. If you are not sure, read the appropriate sections before making your selection.

☒ Life sciences ☐ Behavioural & social sciences ☐ Ecological, evolutionary & environmental sciences

For a reference copy of the document with all sections, see nature.com/documents/nr-reporting-summary-flat.pdf

Life sciences study design

All studies must disclose on these points even when the disclosure is negative.

Sample size	Sample size was chosen based on our ability to process samples and statistical significance was accepted regardless of outcome (i.e. sample sizes were not increased in hopes of obtaining a p value of <0.05)
Data exclusions	No data was excluded from analyses
Replication	All experiments were performed a minimum of twice on independent days.
Randomization	Zebrafish embryos were randomly allocated into experimental groups.
Blinding	Blinding was not relevant or necessary for this study as measurements taken were not subjective.

Reporting for specific materials, systems and methods

We require information from authors about some types of materials, experimental systems and methods used in many studies. Here, indicate whether each material, system or method listed is relevant to your study. If you are not sure if a list item applies to your research, read the appropriate section before selecting a response.

Materials & experimental systems

n/a	Involved in the study	
☐	☒	Antibodies
☒	☐	Eukaryotic cell lines
☒	☐	Palaeontology and archaeology
☐	☒	Animals and other organisms
☒	☐	Human research participants
☒	☐	Clinical data
☒	☐	Dual use research of concern

Methods

n/a	Involved in the study	
☒	☐	ChIP-seq
☒	☐	Flow cytometry
☒	☐	MRI-based neuroimaging

Antibodies

Antibodies used	Cell Signaling 3377 Anti-PH3 ser10 antibody, D2C8 lot number 3. Cell Singling 18338 Phospho-Smad2, E8F3R lot number 9.
Validation	We validated the PH3 antibody with a timecourse, where we showed increased nuclear specific staining during regeneration when there is increased cell division. There are 245 citations on the product webpage. We validated the P-Smad2 antibody by inhibiting tgfb signaling and showing a specific decrease in nuclear staining. There are 50 citations in the product webpage.

Animals and other organisms

Policy information about studies involving animals; ARRIVE guidelines recommended for reporting animal research

Laboratory animals	Danio Rerio. Embryos were too young to identify sex. Wildtype strain used was Tab5.
Wild animals	The study did not involve wild animals.
Field-collected samples	The study did not involve samples collected from the field.
Ethics oversight	All protocols were approved by the NHGRI Animal Care and Usage Committee protocol #G-01-3.

Note that full information on the approval of the study protocol must also be provided in the manuscript.
